# Breast cancer incidence and mortality in population studies of radiation exposure: systematic review and meta-analysis

**DOI:** 10.1038/s41416-026-03503-0

**Published:** 2026-06-16

**Authors:** Ekaterina Chirikova, Cecile M. Ronckers, Mark P. Little, Emma Quinn, Brittany Liu, Richard Hu, Mayela T. Gillan, Brianna Frangione, Tarek Benzouak, Tanvi Srivastava, Lydia B. Zablotska

**Affiliations:** 1 Department of Epidemiology and Biostatistics, School of Medicine, University of California, San Francisco, San Francisco, CA USA; 2 Division of CAYA Cancer Survivorship Research, German Cancer Research Center, Heidelberg, Germany; 3 Division of Childhood Cancer Epidemiology, German Childhood Cancer Registry, Institute for Medical Biostatistics, Epidemiology and Informatics, University Medical Center of the Johannes Gutenberg University, Mainz, Germany; 4 Feinberg School of Medicine, Northwestern University, Evanston, IL USA; 5 Faculty of Health, Science and Technology, Oxford Brookes University, Oxford, UK; 6 Oncology Department, Cumming School of Medicine, University of Calgary, Calgary, AB Canada; 7 CAREX Canada, University of British Columbia, Vancouver, BC Canada; 8 Department of Radiation Oncology, Helen Diller Comprehensive Cancer Center, University of California, San Francisco, San Francisco, CA USA; 9 Environmental Health Sciences Division, School of Public Health, University of California, Berkeley, Berkeley, CA USA; 10 Department of Neuroscience, Carleton University, Ottawa, ON Canada; 11 Faculty of Medicine, McGill University, Montreal, QC Canada

**Keywords:** Breast cancer, Risk factors, Epidemiology

## Abstract

**Background:**

While breast cancer risk from high-dose ionising radiation is known, uncertainties remain about risks at lower doses and risk-modifying factors. We conducted a systematic review and meta-analysis of publications on radiation-associated risk of breast cancer in women.

**Methods:**

We included studies published in 2005–2022 that assessed breast cancer incidence or mortality in women exposed to ionising radiation. Risk of bias was evaluated. Random-effects meta-analyses estimated excess relative risks per gray (ERR/Gy).

**Results:**

Of the 3522 articles screened, 106 met the inclusion criteria; 40 studies provided 44 ERR/Gy estimates. Overall, radiation exposure was associated with increased breast cancer risk (ERR/Gy = 0.56, 95% CI: 0.29–0.83). Between-study heterogeneity (*I*^2^ = 95%) was substantially reduced in subgroup analyses, reaching 5% in low-dose-rate studies. Higher summary ERRs were observed for high dose-rate exposures, moderate (1–5 Gy) doses, childhood exposures, and attained age over 55. Lower but significantly increased risks were estimated for other subgroups and exposure scenarios.

**Conclusions:**

Radiation exposure was associated with a significantly increased risk of breast cancer among women, particularly following high dose-rates, moderate doses, childhood exposures, and older attained age. PROSPERO registration: CRD42021260610.

## Introduction

Breast cancer remains the most frequently diagnosed cancer in women globally, with approximately 2.3 million new cases in 2022—nearly one in four cancer diagnoses worldwide [1]. Breast cancer is the most commonly diagnosed cancer among women in the United States, with an estimated 316,950 new cases expected in 2025, accounting for 32% of all cancers in women [2]. Global trends of breast cancer mortality vary significantly, but breast cancer remains the leading cause of cancer-related deaths in women worldwide, responsible for one in six cancer deaths [1]. In the United States, breast cancer mortality has been declining since 1989 [1], but it remains the leading cause of cancer mortality among women under 50, with an estimated 42,170 deaths projected for 2025 [2]. Well-established risk factors directly influencing breast cancer incidence include increasing age, high-penetrance genes (e.g., BRCA1 and BRCA2), breast density, hormonal factors, and radiation exposure [3]. Understanding these risk factors is critical for developing effective breast cancer prevention strategies for reducing the cancer burden and associated mortality in women.

Radiation-induced carcinogenesis can result from a single exposure to ionising radiation, presumably due to DNA and chromosomal damage in individual cells; however, cumulative and indirect effects also contribute to cancer development, even at low doses [4–7]. Current evidence suggests a linear no-threshold dose-response in relation to radiation-associated risk of breast cancer development [6, 8, 9]. While radiation exposure is a well-established risk factor for breast cancer after high-dose exposures [9–12], some uncertainties remain regarding factors that may modify this risk, including the form of its association with age [11–14], the impact of lifestyle and reproductive factors [10, 15–17], and genetic susceptibility [17–19]. The long-term effects of low-dose exposures, such as environmental and occupational radiation exposures, also require more evidence [20–25].

Previous reviews of radiation-related breast cancer risks covered the literature up to 2005 but were neither systematic nor did they include meta-analysis [9, 26]. New studies have since been published with extended follow-ups of pivotal cohorts, improved dosimetry and study methodology, and evaluation of effect modifiers [10, 18, 22, 27–30]. To reflect these developments, this systematic review and meta-analysis aimed to synthesise current evidence on radiation-associated breast cancer incidence and mortality in women exposed to various dose-rates and doses of radiation while also evaluating potential effect modifiers of this risk and assessing potential threats to validity in the existing literature. Our findings should inform future research directions, provide evidence for public health decisions on radiation safety, and inform clinicians working with patients receiving radiation exposure.

## Methods

### Selection of studies

This systematic review was conducted following the Preferred Reporting Items for Systematic Reviews and Meta-Analysis (PRISMA 2020) guidelines [31], and the protocol was registered in PROSPERO (CRD42021260610) on July 16, 2021.

A search for relevant studies published in or after 2005 with no language restrictions was conducted in PubMed, Embase, CINAHL databases, and the United Nations Scientific Committee on the Effects of Atomic Radiation (UNSCEAR 2006) report [9] on March 30, 2022. The complete list of terms used in the comprehensive search strategy is provided in Supplementary Materials S1. Covidence software [32] was used to select studies that examined the association between radiation exposure and cancer incidence or mortality, and involved human participants. Duplicate studies were removed, and titles and abstracts of selected studies from all databases were independently screened by two reviewers, randomly assigned from among BF, BL, EC, EQ, RH, TB, and TS for each abstract. Any discrepancies between the two reviewers were resolved by discussion or, in case of continued disagreement, adjudicated by a third reviewer (EC).

Full texts of selected studies were obtained for further review. Only full-text articles available in English were included in the review. A detailed list of the inclusion and exclusion criteria for full-text review is presented in Table 1. The outcome of interest was breast cancer incidence or mortality in women only. Ionising radiation was the primary exposure of interest, with different definitions applied depending on the study type. For internal dose-response analyses (i.e., comparisons within the exposed population), only studies reporting individual dose measurements were included. For external comparisons (i.e., between exposed individuals and the general population), studies were included if they reported measures of association without requiring individual dose measurements. For external comparisons in radiation treatment (RT) studies, we included only those where all members of the exposed group were known to receive at least some amount of radiation.

**Table 1:** Inclusion and exclusion criteria for study selection.

Inclusion criteria	Exclusion criteria
• Exposure is ionising radiation • Includes one of the following study outcomes—incidence or mortality due to primary invasive breast cancer or, in the case of radiotherapy exposure, second primary invasive breast cancer. • For studies reporting dose-response analyses (i.e., reporting ERR, RR, HR, or OR per units of radiation dose), radiation exposure is clearly defined and includes mean dose to breasts in gray (Gy) or whole-body dose in sievert (Sv), or equivalent units. If the mean dose is unavailable, the study includes at least the range of doses in the cohort. *Note: This requirement does not apply to studies reporting SIR/SMR*. • For studies reporting only SIR/SMR, radiation exposure is well-defined. Specifically, comparisons are made between those with radiation exposure and those without radiation exposure or with the general population. • Includes information on the size of the female study population at risk, and breast cancer incidence or mortality cases in females. • Any type of population study (e.g., cohort, case-control, cross-sectional). • Includes only the latest update from the same cohort, unless different study methods and/or risk estimation methods were used.	• Studies of breast cancer in males only. • Studies not in humans. • Outcome is second primary breast cancer in the same breast. • Outcome is breast cancer in situ only. • Type of study: case report, case series, cost-effectiveness study, evaluation study, methodological study, survey-based study, descriptive study, ecological study, systematic review, scoping review, meta-analysis. • Type of publication: conference abstract, letter, editorial, commentary, protocol. • Published before 2005. • Full text is not in English. • Previous publications from the same cohort using similar study methods and risk estimation methods.

*ERR* excess relative risk, *RR* risk ratio, *HR* hazard ratio, *OR* odds ratio, *SIR* standardised incidence ratio, *SMR* standardised mortality ratio.

Each full text was independently assessed for eligibility criteria by two reviewers, randomly assigned from among BF, BL, EC, EQ, RH, TB, and TS. Conflicts were resolved through discussion or, if disagreement persisted, adjudicated by a third reviewer (EC or LBZ). Additionally, backward citation chasing was undertaken by two reviewers independently, who manually reviewed the reference lists of all included studies to identify potentially relevant papers not captured by the database search. References judged potentially eligible were uploaded into Covidence, and the same screening process and eligibility criteria used for citations identified through the database search were then applied. Data abstraction on predefined variables was independently performed by two reviewers, randomly assigned from among BF, BL, EC, EQ, MTG, RH, TB, and TS for each included study, and recorded in a Microsoft Excel spreadsheet. Data abstractions were finalised by EC, and the data are provided in Supplementary Materials S2.

### Statistical methods

We synthesised the effect estimates from internal dose-response and external comparison studies separately. The main estimate of radiation risk in internal dose-response studies is the value of the excess relative risk (ERR) per unit of absorbed dose (Gy) of radiation exposure; when study authors reported dose in sieverts (Sv) for low-LET radiation, we treated 1 Sv as equivalent to 1 Gy for comparability. This effect estimate comes from a model that describes the risk imposed by radiation exposure as a multiplicative increment to the excess disease or death risk above the background rate of disease or death [33]. ERR is related to the relative risk (RR) as ERR = RR-1. We converted all reported effect estimates to ERR per 1 Gy (ERR/Gy) for consistency, following the assumption that odds ratios (OR) approximate RR. For our meta-analysis, estimates were included with their corresponding 95% confidence intervals (CI). In cases with a missing lower CI bound (i.e., the model did not converge) or 90% CI, we applied the Wald method to approximate 95% CI assuming a normal distribution of the estimate [34].

The estimates of radiation risk in external comparison studies are the values of the standardised incidence or mortality ratios (SIR and SMR). These measures reflect the occurrence of disease or death in the population, all members of which were exposed to at least some amount of radiation, relative to what would be expected in the general or, in some cases, unexposed population. Our analysis included these estimates with their corresponding 95% CIs. In cases where CIs were missing, we reconstructed the 95% CIs using the formula for the exact fiducial interval [35, 36].

In addition to effect estimates, we extracted other relevant information from the studies for use in subsequent analyses, including mean, median, and maximum radiation dose (i.e., peak exposure), dose type (breast, whole body, other), maximum radiation dose-rate, age at exposure, age at risk, follow-up time, concomitant chemotherapy (for studies of RT), exposure group (A-bomb, environmental, occupational, diagnostic, RT for cancer and non-cancer disease studies), and outcome type (incidence, mortality).

To generate summary measures of the association between radiation exposure and breast cancer outcome, we fit random effects models using the restricted maximum likelihood method, which provides unbiased estimates of variance [37, 38]. In addition to the crude summary estimate, we also evaluated effect modification by factors such as outcome type, exposure group, maximum radiation dose-rate (low: <5 mGy/h, high: ≥5 mGy/h), dose type, maximum radiation dose, as well as age at exposure, age at risk, and concomitant chemotherapy (for studies of RT), where available. Each potential effect modifier was evaluated in a separate model rather than jointly, because the limited number of studies did not allow for stable estimates in models with multiple modifiers. The Knapp-Hartung adjusted *F*-test was used to evaluate the joint significance of risk modifier coefficients [39, 40].

To evaluate residual heterogeneity, we applied Cochran’s *Q* test [41]. To further quantify the proportion of total variation attributable to heterogeneity across studies, we calculated the *I*² statistic, as described by Higgins and Thompson [42]. The *I*^2^ values are presented as percentages, where values close to 0% indicate minimal between-study heterogeneity compared to within-study variance. In comparison, values near 100% suggest that between-study heterogeneity accounts for most of the observed variability. Confidence intervals for the *I*^2^ statistic were estimated using the approach outlined by Knapp and Hartung [40]. We also conducted a sensitivity analysis by sequentially excluding groups of studies based on exposure type—first removing studies of survivors of atomic bombings (A-bomb), followed by those involving RT—and assessed the resulting changes in heterogeneity. Given prior concerns that the 2007 study by Cardis et al. [43] may have led to an overestimation of pooled radiation risks [44], we performed a sensitivity analysis excluding this study to evaluate its influence on the meta-excess relative risk.

While the summary measures were calculated for studies reporting ERR, they were not calculated for studies reporting SIR/SMR, as combining estimates with different external comparisons was deemed impractical due to significant heterogeneity.

To assess publication bias, we used Egger’s test and funnel plots [45, 46]. An inverted symmetrical funnel shape of the plot suggests minimal publication bias. We also used Duval and Tweedie’s trim-and-fill method to evaluate the potential impact of publication bias on the excess relative risk estimates [47].

We assessed the risk of bias for each study following the Office of Health Assessment and Translation (OHAT) guidelines [48]. Each study was evaluated by two independent reviewers, randomly assigned from among BL, EQ, MTG, RH, TB, and TS, using a standardised protocol. Discrepancies were resolved through discussion or with input from LBZ, who also reviewed the final assessments. A four-point rating scale was used to evaluate selection bias, confounding, inclusion of important effect modifiers, attrition/data exclusion, exposure classification bias, outcome assessment, outcome reporting, and other threats to internal validity. For each study, an average risk of bias score was calculated. Studies with scores below three were considered lower quality and were filtered out for certain sensitivity analyses.

Further details on statistical methods are given in the Supplementary Materials S1. All statistical tests were two-sided, with *α* = 0.05. All statistical analyses were performed in R Statistical Software (v 4.3.3) [49], using the metafor [39] and ggplot2 [50] packages.

## Results

**Fig. 1:**
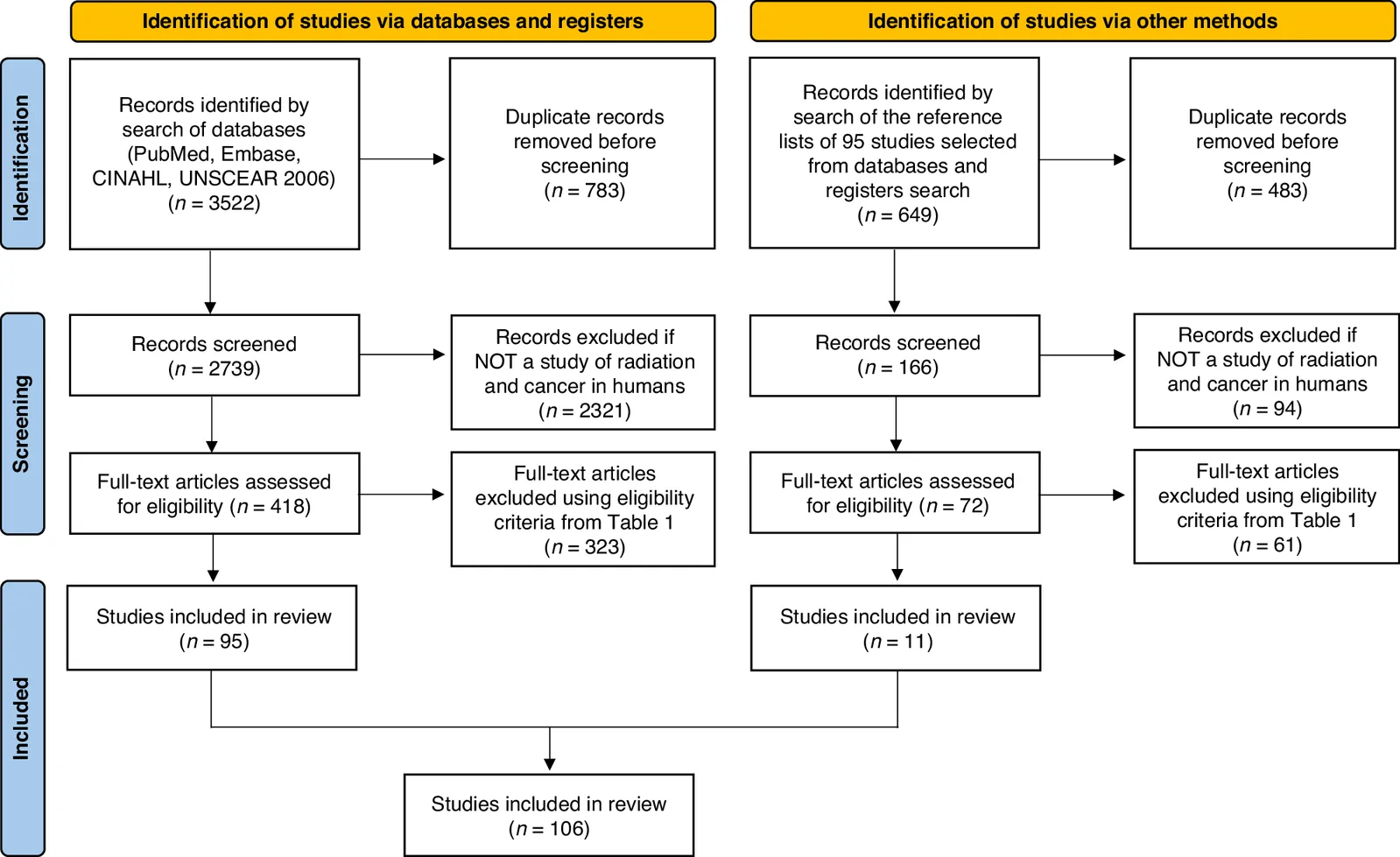
PRISMA 2020 flow diagram of study selection. CINAHL Cumulative Index to Nursing and Allied Health Literature, UNSCEAR United Nations Scientific Committee on the Effects of Atomic Radiation.

Following the screening and selection processes, 106 studies were included in the final review by consensus, from a total of 4171 studies (3522 identified through database searches and 649 identified through reference list screening) (Fig. 1). After excluding duplicates (1266, 30%) and studies that did not examine radiation and cancer in humans (2415, 58%), we conducted a full-text review of the remaining 490 studies. We further excluded 384 (78%) studies due to: reporting an irrelevant outcome (i.e., not breast cancer; *n* = 73, 15%), not reporting a measure of association between radiation exposure and breast cancer (*n* = 66, 13%), presenting projected rather than observed outcomes (e.g., modelling studies; *n* = 65, 13%), and other reasons (Supplementary Materials S1 and Table S1.1). Of the 106 included studies, 31 reported ERR only, 66 reported SIR/SMR only, and 9 reported both types of estimates. Of the 40 studies reporting ERR and included in the meta-analysis, 19 used dose-to-breast, 7 used colon or other organ dose, and 14 used full body dose measurements. Among these same studies, 29 reported incidence outcomes, 9 reported mortality outcomes, and 2 reported both. Because of the limited number of studies focusing on mortality, studies reporting incidence and mortality outcomes were analysed together, rather than in separate subgroup analyses.

Risk of bias analysis indicated that 68 internal dose-response and external comparison studies (64%) received a score of 3.0 or higher, which we interpreted as indicative of higher study quality and minimal risk of bias. Detailed results for each risk of bias domain by study are available in Supplementary Materials S4. The most common source of bias was selection bias, inherent to RT studies (due to survival bias) and occupational exposure studies (due to the healthy worker effect). Inadequate adjustment for confounding factors was another frequent reason for lower scores, especially in environmental exposure studies [51–56]. Many included studies faced substantial challenges in accurately estimating radiation dose, with common issues including reliance on proxy indicators of exposure (e.g., occupational title, geographic residence, or effective dose) [16, 29, 52, 57, 58], uncertainties in dosimetry [25, 59], and incomplete treatment records [14, 60].

Due to the large number of studies, detailed information on each, including full citations, adjustment sets, and other study characteristics, is provided in Supplementary Materials S2.

### Results of meta-analysis

#### Main effects

**Fig. 2:**
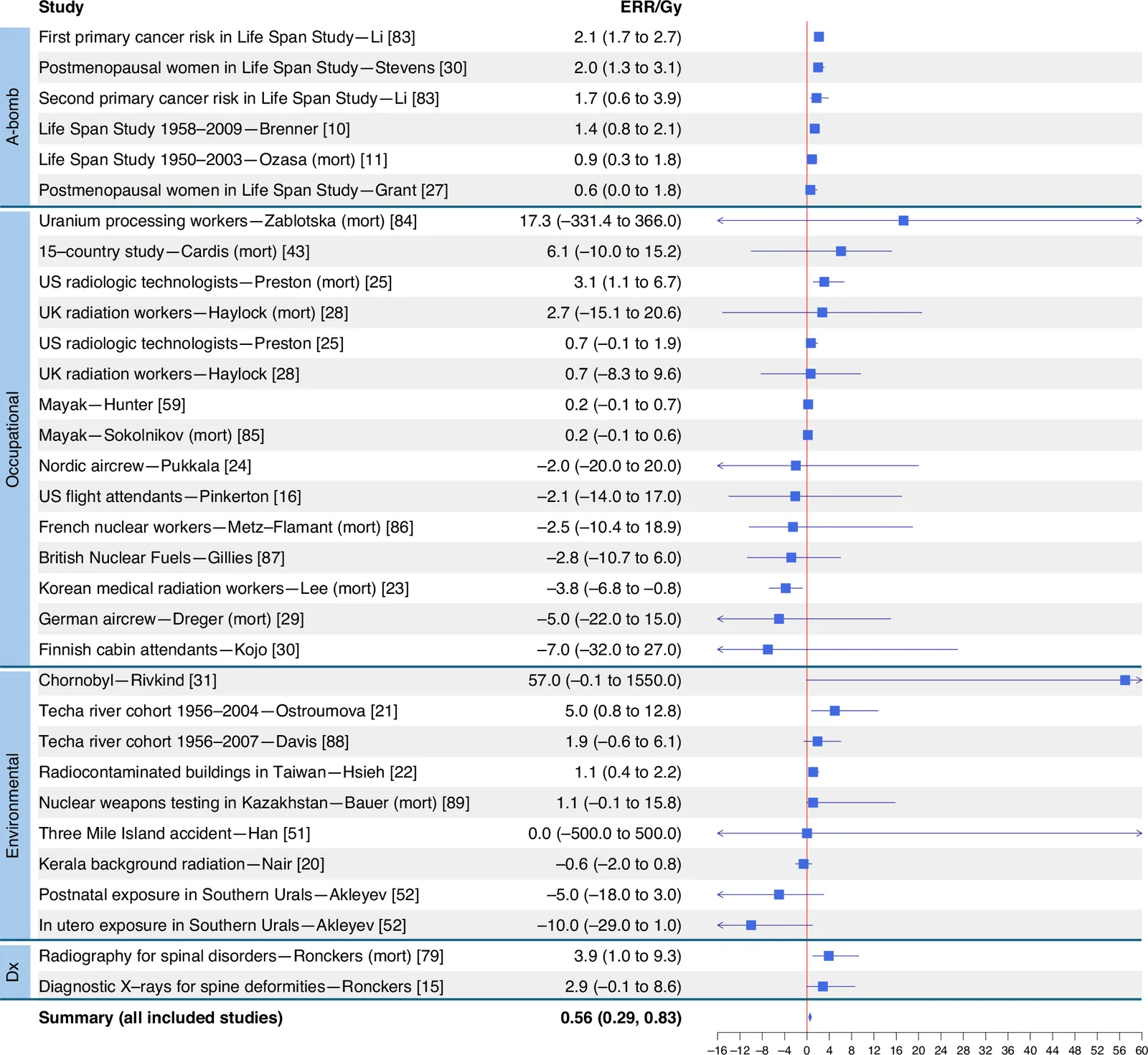
Forest plot showing excess relative risks with confidence intervals for breast cancer by four radiation exposure groups (excluding radiation treatment studies). References are shown in square brackets. ERR excess relative risk, Gy gray, Dx diagnostic exposure, A-bomb studies of atomic bomb survivors. The summary estimate (shown below the forest plot) is calculated from all five exposure groups, including radiation treatment studies. Mortality studies marked with “(mort)”. Heterogeneity for summary estimate: *p* value for residual heterogeneity <0.001, *I*^2^ = 95.12 (95% CI 88.27, 98.40) [83–89].

**Fig. 3:**
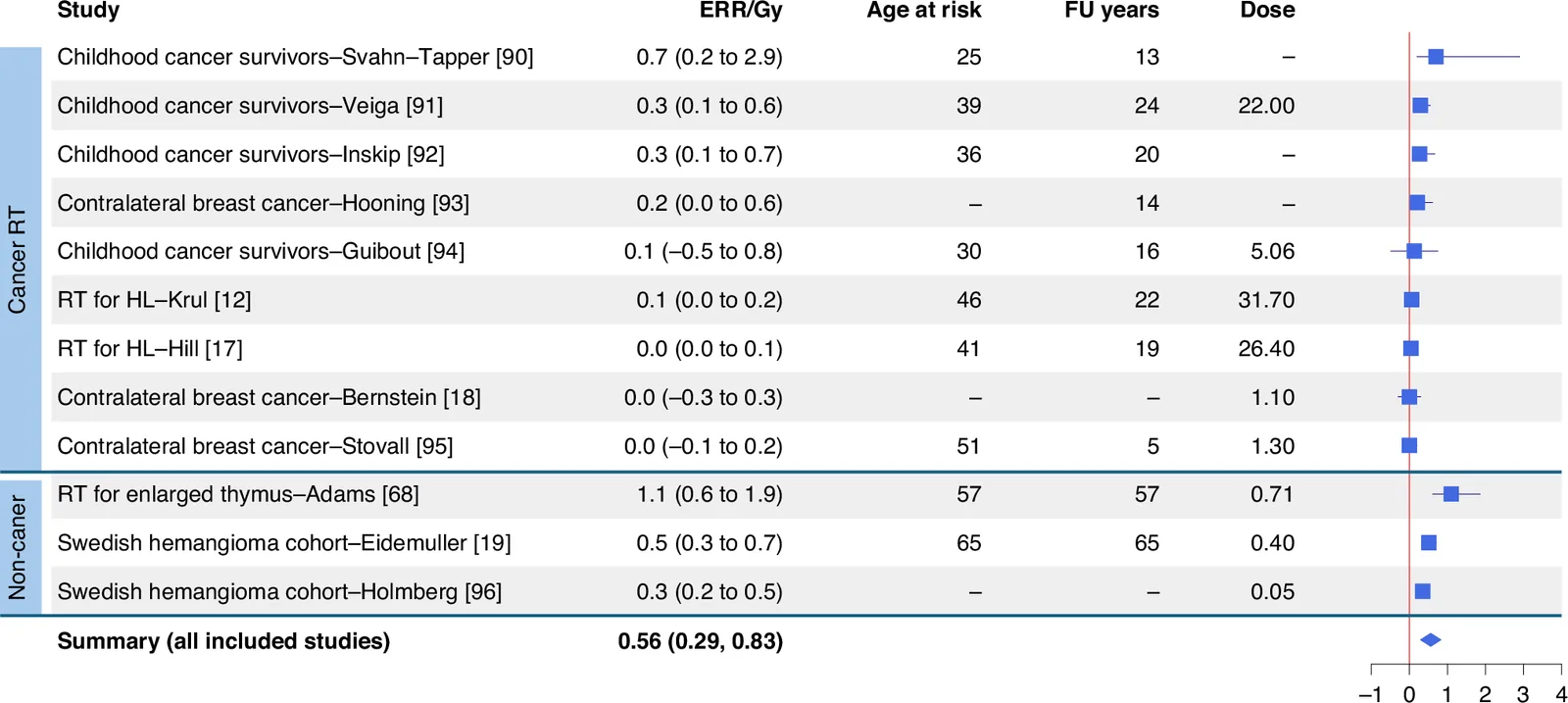
Forest plot showing excess relative risks with confidence intervals for breast cancer for radiation treatment studies. References are shown in square brackets. ERR excess relative risk, Gy gray, FU follow-up, RT radiation treatment, HL Hodgkin’s lymphoma. The summary estimate (shown below the forest plot) is calculated from all five exposure groups, including radiation treatment studies. The dose column shows the mean or median dose in gray. Heterogeneity for summary estimate: *p* value for residual heterogeneity <0.001, *I*^2^ = 95.12 (95% CI 88.27, 98.40) [90–96].

Radiation exposure was associated with a statistically significant increased meta-excess relative risk of breast cancer in the synthesis of 44 estimates from 40 studies that reported ERR estimates (Table 2). Specifically, for each 1 Gy increase in radiation exposure, the risk of breast cancer increased by 56% above the background breast cancer rate (95% CI: 29–83%). Forest plots in Figs. 2, 3 provide a graphical summary of the ERRs from all included studies. The between-study heterogeneity was statistically significant, with *I*^2^ = 95% (95% CI: 88–98%), indicating substantial heterogeneity (Table 2).

**Table 2:** Meta-analysis of excess relative risk for breast cancer as a result of radiation exposure

	No. of study endpoints	Meta excess relative risk/Gy (95% CI)	*p* value for risk modification^a^	*p* value for residual heterogeneity^b^	*I*^2^ (%) (Hartung-Knapp 95% CI)^c^
Overall effect	44	0.56 (0.29, 0.83)	–	<0.001	95.12 (88.27, 98.40)
Effect modification by outcome^d^					
Mortality	11	0.53 (−0.30, 1.37)	<0.001	<0.001	95.42 (88.81, 98.52)
Incidence	33	0.56 (0.27, 0.86)			
Effect modification by exposure group (dose range)					
A-bomb studies (≤4 Gy)	6	1.55 (1.19, 1.92)	<0.001	0.05	26.45 (0.00, 96.55)
Occupational exposure (≤5 Gy)	15	0.21 (−0.09, 0.51)			
Environmental exposure (≤5 Gy)	9	0.71 (−0.14, 1.56)			
Diagnostic exposure (≤1.7 Gy)	2	3.40 (−0.06, 6.87)			
Radiation treatment for cancer (≤80 Gy)	9	0.09 (−0.002, 0.19)			
Radiation treatment for non-cancer disease (≤35 Gy)	3	0.47 (0.28, 0.65)			
Effect modification by dose type (dose range)					
Breast (≤80 Gy)	20	0.47 (0.20, 0.75)	<0.001	<0.001	93.30 (81.64, 98.13)
Whole-body (≤4 Gy)	16	0.18 (−0.45, 0.81)			
Other (colon, stomach, unknown) (≤1 Gy)	8	1.34 (0.65, 2.03)			
Effect modification by exposure rate					
Low dose-rate, <5 mGy/h	28	0.45 (−0.03, 0.93)	<0.001	<0.001	95.02 (87.82, 98.37)
High dose-rate, ≥5 mGy/h	16	0.61 (0.28, 0.95)			
Effect modification by maximum dose^e^					
Maximum dose ≤0.5 Gy	15	0.27 (−0.68, 1.21)	<0.001	<0.001	92.78 (81.29, 98.06)
Maximum dose >0.5 Gy to ≤1 Gy	5	0.15 (−2.89, 3.19)			
Maximum dose >1 Gy to ≤5 Gy	12	1.07 (0.67, 1.46)			
Maximum dose >5 Gy	12	0.26 (−0.03, 0.55)			
Effect modification by age at exposure group^f^					
Childhood	11	0.39 (0.27, 0.50)	<0.001	0.22	1.02 (0.00, 96.53)
Adulthood	18	0.05 (−0.09, 0.19)			
Effect modification by age at risk group^g^					
25–44 years	5	0.16 (−0.01, 0.32)	0.006	0.11	35.38 (0.00, 99.35)
45–54 years	5	0.04 (−0.14, 0.22)			
55–70 years	4	0.54 (0.25, 0.83)			

*Gy* gray, *CI* confidence interval.

^a^Each model is assessing how the excess relative risk of breast cancer per Gy of radiation exposure differs across various factors, i.e., exposure groups, radiation types, maximum doses, outcomes, age at exposure groups, and age at risk groups. Each factor was evaluated in a separate model rather than jointly.

^b^Residual heterogeneity was assessed using Cochran’s *Q* statistic.

^c^*I*^2^ statistic shows the proportion of total variation attributable to heterogeneity between studies vs. within studies.

^d^Indicator variable denoting whether the study reported an incidence or mortality outcome was included to assess this effect modification.

^e^Maximum dose reflects peak exposure rather than cumulative dose.

^f^Only 28 studies reported this information. Childhood exposure is defined as exposure occurring up to and including ages 18–21. Adulthood exposure is defined as exposure occurring after age 18–21.

^g^Only 14 studies reported this information. Age at risk, or attained age, is a study population characteristic.

There was no evidence of publication bias based on visual inspection of funnel plots or results from formal statistical tests (Supplementary Materials S1, Supplementary Fig. S1.1A, B, all tests >0.5). The bias-corrected meta-excess relative risk per Gy, estimated using the Tweedie trim-and-fill method, was very similar to the original estimate with no meaningful change in the confidence interval (not shown).

#### Effects of risk-modifying factors

The magnitude of meta-excess relative risk per Gy varied significantly across a number of risk factors, including exposure group and dose type, dose-rate, maximum dose, outcome type, age at exposure, and age at risk (all *p* < 0.001 except the last one, *p* = 0.006) (Table 2). Summary ERR estimate for studies reporting breast cancer incidence overall showed a statistically significant risk increase (ERR/Gy = 0.56, 95% CI: 0.27–0.86) with a similar but not statistically significant estimate for mortality-based studies (ERR/Gy = 0.53, 95% CI: −0.30 to 1.37).

In analyses by exposure group, the highest estimate was observed for A-bomb survivors’ studies (ERR/Gy = 1.55, 95% CI: 1.19–1.92) (Table 2), with lower but still statistically significant summary estimates for studies of RT used to treat non-cancer conditions (ERR/Gy = 0.47, 95% CI: 0.28–0.65). Summary ERR estimates for other exposure groups were positive, but not statistically significant.

The majority of studies used breast dose in the analysis. We observed significant heterogeneity in risk estimates across studies using breast dose, whole-body dose, or doses to adjacent organs (*p* < 0.001), with the highest risks observed in the latter group (Table 2).

In analyses by exposure rate, a higher summary ERR was observed for studies involving high dose-rate exposure compared to those with low dose-rate exposure (Table 2). The difference in summary ERRs was also observed by maximum radiation dose (i.e., peak exposure), with meta-excess relative risk being the highest for exposures 1–5 Gy (ERR/Gy = 1.07, 95% CI: 0.67–1.46), whereas other groups of exposures, ≤1 Gy and >5 Gy, were associated with a lower ERRs and did not reach statistical significance (Table 2).

Summary ERR estimate for studies of exposure during childhood showed a statistically significant risk increase (ERR/Gy = 0.39, 95% CI: 0.27–0.50). In comparison, summary ERR for studies of exposure during adulthood was lower and not statistically significant (ERR/Gy = 0.05, 95% CI: −0.09 to 0.19) (Table 2).

Lastly, across the fourteen studies that reported age at risk, meta-excess relative risk was the highest for those aged 55–70 years at cancer diagnosis or death (ERR/Gy = 0.54, 95% CI: 0.25–0.83), while younger groups showed lower, non-significant risks (Table 2).

#### Subgroup and Sensitivity Analyses

When groups of studies, specifically A-bomb and those involving RT, were sequentially excluded from the meta-regression, between-study heterogeneity, as measured by the *I*^2^ statistic, decreased markedly—from 95% to 67%, and then to 0%, respectively—suggesting that these study groups were the primary contributors to heterogeneity (Supplementary Materials S1, Table S1.2). At the same time, the estimate of meta-excess relative risk of breast cancer decreased by 52% (from 56%, 95% CI: 29 to 83%, to 27%, 95% CI: 1 to 53%) after excluding 6 A-bomb studies and 12 RT studies (Supplementary Materials S1, Table S1.2).

The results of our meta-analysis restricted to studies with low dose-rate exposures (Table 3) showed evidence of risk modification by dose type, maximum dose, and age at exposure, also observed in the full analysis. One notable difference was that in the analysis by maximum radiation dose (i.e., peak exposure), summary ERR was significantly increased only for exposures >5 Gy (ERR/Gy = 0.42, 95% CI: 0.23–0.62). Between-study heterogeneity was relatively low in this subgroup of studies, ranging from 2% to 5%, indicating that studies of low-dose-rate exposures represented a relatively homogeneous set of studies.

**Table 3:** Meta-analysis of excess relative risk for breast cancer as a result of radiation exposure, restricted to a specific group of studies

	No. of study endpoints	Meta excess relative risk/Gy (95% CI)	*p* value for risk modification^a^	*p* value for residual heterogeneity^b^	*I*^2^ (%) (Hartung-Knapp 95% CI)^c^
Low dose-rate studies, <5 mGy/h^d^					
Overall effect	28	0.38 (0.22, 0.54)	–	0.22	5.33 (0.00, 95.93)
Effect modification by dose type (dose range)					
Breast (≤80 Gy)	7	0.45 (0.27, 0.62)	<0.001	0.24	2.49 (0.00, 93.71)
Whole-body (≤4 Gy)	16	0.22 (−0.07, 0.50)			
Other (colon, stomach, unknown) (≤1 Gy)	5	0.09 (−1.37, 1.55)			
Effect modification by maximum dose^e^					
Maximum dose ≤0.5 Gy	15	0.32 (−0.57, 1.20)	0.001	0.15	1.82 (0.00, 93.16)
Maximum dose >0.5 Gy to ≤1 Gy	5	0.22 (−3.17, 3.62)			
Maximum dose >1 Gy to ≤5 Gy	6	0.28 (−0.02, 0.59)			
Maximum dose >5 Gy	2	0.42 (0.23, 0.62)			
Effect modification by age at exposure group^f^					
Childhood	6	0.43 (0.27, 0.59)	<0.001	0.31	1.82 (0.00, 93.16)
Adulthood	16	0.21 (−0.07, 0.49)			
Radiation treatment studies					
Overall effect	12	0.22 (0.06, 0.37)	–	<0.001	87.53 (69.59, 97.95)
Effect modification by treatment combination					
All had RT only	3	0.46 (0.27, 0.65)	<0.001	0.08	41.63 (0.00, 95.67)
Some had chemotherapy	9	0.08 (−0.005, 0.17)			
Effect modification by exposure rate^g^					
Low dose-rate	2	0.42 (0.25, 0.59)	<0.001	0.20	20.30 (0.00, 90.78)
Fractionated moderate/high dose-rate	9	0.07 (0.01, 0.13)			
Acute moderate/high dose-rate	1	1.10 (0.31, 1.89)			

*Gy* gray, *CI* confidence interval, *RT* radiation treatment.

^a^Each model is assessing how the excess relative risk of breast cancer per Gy of radiation exposure differs across various factors, i.e., exposure groups, radiation types, maximum doses, outcomes, age at exposure groups, and age at risk groups. Each factor was evaluated in a separate model rather than jointly.

^b^Residual heterogeneity was assessed using Cochran’s *Q* statistic.

^c^*I*^2^ statistic shows the proportion of total variation attributable to heterogeneity between studies vs. within studies.

^d^The estimates for the low dose-rate studies slightly vary due to different estimation methods: ERR = 0.44 (95% CI −0.04, 0.92) is derived from a model including all studies and allowing for effect modification by dose-rate, while ERR = 0.38 (95% CI 0.22, 0.53) is derived from a model including only the subgroup of low dose-rate studies.

^e^Maximum dose reflects peak exposure rather than cumulative dose.

^f^Only 21 studies reported this information. Childhood exposure is defined as exposure occurring up to and including ages 18–21. Adulthood exposure is defined as exposure occurring after age 18–21.

^g^Low dose-rate RT: delivers radiation continuously at a low rate, ≤2 Gy per hour, fractionated moderate/high dose-rate RT: delivers radiation at a higher rate (>12 Gy per hour) in multiple sessions (fractions), acute moderate/high dose-rate RT: administers a high dose of radiation in a single or few sessions. The fractionated group comprises studies with a wide range of mean breast doses, varying from 1 to 32 Gy.

In the subgroup of RT studies (Table 3), the summary ERR was significantly higher (*p* < 0.001) for studies involving patients who received RT alone (all involving treatment for non-cancer conditions; ERR = 0.46, 95% CI: 0.27–0.65) compared to those who also received chemotherapy (cancer treatment, fractionated exposure; ERR = 0.08, 95% CI: −0.005 to 0.17).

In the meta-analysis restricted to studies with minimal risk of bias (Supplementary Materials S1, Supplementary Table S1.3), the trends in summary estimates were generally consistent with those described above, but many estimates were lower, including the overall meta-excess relative risk (ERR/Gy = 0.46, 95% CI: 0.20–0.73). Additionally, the meta-excess relative risk remained unchanged after exclusion of the Cardis study (ERR/Gy = 0.56, 95% CI: 0.29–0.83) [43].

#### Summary of external comparison studies

Among studies reporting standardised risk ratios (Supplementary Figs. 1.2 and 1.3), those focused on A-bomb survivors and most studies of RT consistently showed increased breast cancer risk among radiation-exposed populations. In A-bomb studies, standardised mortality ratios (SMRs) ranged from 1.59 to 1.65 and were statistically significant, with narrow confidence intervals (Fig. 1.2). However, the magnitude of standardised risk ratios varied widely across the 35 RT studies included in this review owing to the differences in age and size of the cohort and attained age of the cases. Positive associations ranged from an SMR of 1.2 (95% CI: 1.0–1.4) for breast cancer following RT for thyroid cancer (same estimate and 95% CI for non-RT group) [61] to an exceptionally high SMR of 72.3 (95% CI 26.5–157.3) for Hodgkin’s lymphoma survivors [62]. Five of the 35 studies (14%), likely involving low doses to the breast, although not reported, found either no association or inverse associations between radiation exposure and breast cancer risk (Fig. 1.3). These included studies of subsequent cancer risk following RT for thyroid cancer [63], endometrial cancer [64], and non-Hodgkin lymphoma (NHL) [65], as well as studies of RT for non-malignant conditions such as tinea capitis [66] and benign gynaecologic disorders [67].

Studies of environmental, occupational, and diagnostic radiation exposure showed substantial heterogeneity (Fig. 1.2). Estimates of standardised incidence and mortality ratios varied both in magnitude and direction. Most positive associations in these studies fell within the range of 1 to 1.5.

## Discussion

Here we presented the results of the first systematic literature search with meta-analysis, identifying 106 studies published from 2005 to 2022 that examined the association between radiation exposure and breast cancer risk. From these, 40 studies reporting 44 ERR estimates were included in the meta-analysis, showing a significant increase in breast cancer risk due to radiation, where each 1 Gy increase in exposure was associated with a 56% increase in meta ERR above the background rate of breast cancer. Substantial heterogeneity was observed, primarily due to studies of atomic bomb survivors and radiotherapy. But heterogeneity was considerably reduced when these studies were excluded, with an accompanying reduction in the meta-analytic risk estimate. Risk estimates varied by dose-rate, dose type, maximum dose, age at exposure, age at risk, exposure group, and outcome type, with higher ERRs linked to high dose-rates, moderate doses (1–5 Gy), childhood exposures, and older attained age. Higher ERRs were also observed in studies of breast cancer incidence than in studies of breast cancer mortality. This difference may reflect the predominance of occupational studies among the mortality analyses, which typically involve low-dose-rate exposures (Supplementary Materials S1, Supplementary Table S1.4), and possible underascertainment in mortality studies if breast cancer is not consistently recorded as the underlying cause of death on death certificates.

Higher summary ERRs were observed for moderate maximum doses (1–5 Gy), high dose-rate exposures, childhood exposures, and attained age 55–70 years. While the latter effects have been reported for other cancers, the observation of a non-linear dose-response has not been described previously. Maximum dose reflects peak exposure rather than cumulative dose, which is the metric typically used to estimate ERR/Gy. Therefore, stratification by maximum dose may not directly correspond to the underlying dose–response model. The highest ERR was observed in the 1–5 Gy category (12 studies), whereas the ≤0.5 Gy (15 studies), 0.5–1 Gy (5 studies), and >5 Gy (12 studies) groups showed lower estimates that were largely non-significant. The wide confidence interval in the 0.5–1 Gy category likely reflects the small number of contributing studies. The lower ERR in the >5 Gy group may similarly reflect heterogeneity in high-dose exposure contexts, potential survivor effects, or differences in exposure type and study design. Accordingly, the apparent non-linearity across maximum dose categories should be interpreted cautiously, as it may arise from heterogeneity and statistical instability rather than a true departure from linearity.

Several of the included studies reported on additional potential risk-modifying factors not previously discussed in this review, such as genetic susceptibility, biomarkers, reproductive and hormonal influences, and concurrent chemotherapy (summarised in Supplementary Materials S1, Supplementary Table S1.5). While data on genetic factors are limited, the WECARE study reported elevated ERR/Gy among ATM variant carriers [18]. Evidence on the role of family history was inconsistent [15, 17, 19]. There is also limited evidence from the LSS of a mediating role of bioavailable estradiol in postmenopausal women [27, 30] and ERR/Gy being highest among females exposed around menarche [10]. Otherwise, most studies reported no significant effect modification by reproductive variables, including menopausal status, age at menopause, parity, age at first birth, or hormonal use [10, 12, 15, 68], although with a few exceptions [16, 17].

This study has several strengths. Compared to the previous narrative review by Ronckers et al. [26], which provided a broad summary of radiation-associated breast cancer risk based on selected epidemiologic and animal studies, our study represents the first systematic review and meta-analysis to quantitatively synthesise risk estimates across a comprehensive set of studies. The previous review highlighted key modifiers such as age at exposure, parity, and menopausal status. Our analysis not only confirmed the importance of established modifiers but also provided quantitative summary estimates by categories of exposure type, dose type, and demographic factors. Our review also incorporates evidence from one of the three large cohort studies published since the previous review [10, 69, 70], which have provided consistent support for heightened breast susceptibility to radiation during puberty, with risk per gray peaking around menarche.

In addition to being the first systematic review and meta-analysis to quantitatively evaluate the association between radiation exposure and breast cancer risk, our study has the following strengths: (1) the study protocol was pre-registered online to promote transparency and reduce potential bias, (2) we employed robust statistical methods that accounted for heterogeneity across studies and conducted extensive sensitivity analyses, (3) findings were consistent even when analyses were restricted to high-quality studies, underscoring the reproducibility of the results, (4) no evidence of publication bias was detected, (5) all screening steps were conducted independently by two reviewers, with disagreements resolved by a third reviewer, and data abstraction was similarly performed by two independent reviewers and confirmed by a third, ensuring consistency and methodological rigour throughout the review process.

A key limitation of this study is the high between-study heterogeneity of the combined estimate, as reflected in the low *p* value of the Cochrane test and the high *I*^2^ statistic, which complicates the causal interpretation of the findings. However, heterogeneity declined considerably after exclusion of the A-bomb and RT studies, and it was also substantially lower in subgroup analyses of low-dose-rate and RT studies. These lower *I*^2^ values should nonetheless be interpreted cautiously, as the upper bounds of their 95% CIs often exceeded 90%, reflecting the Hartung-Knapp method’s tendency to overstate uncertainty in small samples. Such heterogeneity underscores the inherent complexity of radiation-related risk, which depends not only on dose but also on dose-rate, exposure duration, age at exposure, and study-specific characteristics such as population, follow-up period, and covariate adjustment.

Differences in dose metrics across studies represent an important source of uncertainty in our study. While many studies used breast dose, others relied on doses to surrogate organs or whole-body/effective dose. Treating these measures as directly comparable in the overall meta-analysis leads to discounting differences in organ-specific energy deposition and radiation tissue weighting, and may introduce systematic measurement error. Such misclassification could contribute to the heterogeneity observed across studies and may bias ERR/Gy estimates, although the direction and magnitude of this bias are difficult to predict. In meta-regression analyses, risk estimates significantly varied by dose metric type, suggesting that dosimetric differences partly explain between-study heterogeneity. However, sensitivity analyses restricted to studies with breast dose estimates (20 studies) yielded results broadly similar to the overall estimate (ERR/Gy = 0.44, 95% CI: 0.17–0.71 vs. 0.56, 95% CI: 0.29–0.83, respectively), supporting the robustness of the main findings. Nonetheless, residual uncertainty in dose comparability across studies should be considered when interpreting our results.

Notably, many studies, particularly those involving RT, lacked key data such as mean age at risk, follow-up time, and radiation dose [71], limiting our ability to fully assess or adjust for these factors. In RT studies, the use of single summary estimates is particularly complicated by confounding from co-administered chemotherapy agents [72, 73] and shared aetiologic factors between primary and subsequent malignancies [74, 75], making it difficult to isolate the independent effect of radiation. Additional challenges and limitations related to radiation risk assessment are detailed in Supplementary Materials S1, Supplementary Discussion.

A fundamental assumption in most meta-analyses is that studies and endpoints are statistically independent, but this assumption does not hold for several studies in the current paper. For instance, the studies of mortality and incidence in the atomic bomb survivors, in the UK nuclear workers, and in the US radiological technologists are based on the same or similar cohorts and thus are not independent. Ignoring dependence can bias point estimates and their estimated standard errors. There are meta-analysis methods that can account for this dependence [76, 77], but they require data that were not available. A sensitivity analysis excluding mortality studies of the same cohorts as incidence studies yielded results similar to the overall estimate (ERR/Gy = 0.53, 95% CI: 0.25–0.82 vs. 0.56, 95% CI: 0.29–0.83, respectively), supporting the robustness of the main findings.

Our findings have notable implications for clinicians. The increased breast cancer risk associated with moderate radiation doses, especially when exposure occurs during vulnerable life stages, highlights the importance of minimising unnecessary radiation in medical therapeutics, particularly among younger patients. In view of a broad spectrum of late effects, this is common practice for (radiotherapy) treatment planning among children and adolescents [78]. While the effects of radiation exposure from diagnostic imaging procedures are of particular interest because of the growing number of CT scans, we are not able to provide a meaningful summary of evidence as only two reports from a single study on diagnostic radiation were included in this review [15, 79]; large-scale studies of CT scans to date lack sufficient follow-up and case numbers to assess breast cancer risk directly [80].

Despite the progress made in quantifying radiation-associated breast cancer risk in women exposed to various types of radiation under different exposure scenarios, several unanswered questions remain. Future research should focus on pooling individual-level data [81, 82] to better understand how factors, such as genetic susceptibility, hormonal factors, and co-exposures, interact with radiation to influence breast cancer risk. High-quality, large longitudinal studies with detailed exposure reconstruction and measured covariates will be critical to disentangle these complex relationships. In particular, the role of low-dose and fractionated exposures, as commonly encountered in medical and occupational settings, warrants further investigation. Additionally, the potential for gene-environment interactions involving radiation-responsive genes remains an important area for future inquiry, but is hampered by a lack of collection of biological samples in longitudinal radiation epidemiology studies.

## Conclusions

This systematic review and meta-analysis provide robust quantitative evidence that radiation exposure is significantly associated with increased risk of breast cancer in women, particularly at moderate doses and high dose-rates. Our findings underscore the importance of exposure timing and characteristics, with acute exposures during sensitive developmental windows posing greater risk. These insights support more informed decision-making for clinicians regarding radiation use and safety. As an example, patients exposed to radiation in clinical settings should be evaluated for radiation effects if breast tissue was exposed. Continued research is needed to clarify dose-response relationships at low exposure levels, explore gene-environment interactions, and improve individual-level risk prediction.

## Supplementary information


Supplement S1
Supplement S2
Supplement S3
Supplement S4
PRISMA 2020 checklist


## Data Availability

All data used in the article are provided in the online Supplementary S3.

## Code availability

R code is provided in Supplementary Materials S3.
