# Supplementary material for: Breast cancer incidence and mortality in population studies of radiation exposure: systematic review and meta-analysis
Source: Br J Cancer. 2026 Jun 16;135(6):941–52. doi: 10.1038/s41416-026-03503-0 (PMC13534715; doi:10.1038/s41416-026-03503-0)
Supplement: Supplementary file 1 [file 41416_2026_3503_MOESM1_ESM.docx]

**SUPPLEMENTAL MATERIALS**

**SUPPLEMENTAL METHODS**

*Details of database search*

Search for relevant studies published in or after 2005 with no language restrictions was conducted in PubMed, Embase, CINAHL databases, and the United Nations Scientific Committee on the Effects of Atomic Radiation (UNSCEAR 2006) [1] report on March 30, 2022. The literature searches produced 3,522 articles which were uploaded to Covidence on March 30, 2022. As an example, presented below is the search script used for PubMed search.

Search script:

(((breast neoplasms[mh] OR (neoplasms[mh] AND breast*[tiab])) AND (atomic bomb survivors[tiab] OR A-bomb survivors[tiab]))

OR

((breast neoplasms[mh] OR (neoplasms[mh] AND breast*[tiab]))

AND (neoplasms, second primary[mh] OR second breast cancer*[tiab] OR subsequent breast cancer*[tiab] OR secondary breast cancer*[tiab] OR (subsequently[tiab] AND breast cancer*[tiab]) OR childhood cancer survivor*[tiab])

AND (dose-response relationship, radiation[mh] OR radiation, ionizing[mh] OR radiotherapy/adverse effects[mh] OR X-ray therapy/adverse effects[mh] OR radiation injuries[mh] OR radiotherapy dosage[mh] OR radiation dose[tiab] OR radiotherapy[tiab] OR chest radiation[tiab] OR chest irradiation[tiab] OR radiation-based therapy[tiab] OR therapeutic radiation[tiab]))

OR

((breast neoplasms[mh] OR (neoplasms[mh] AND breast*[tiab]))

AND (neoplasms, radiation-induced[mh] OR (cancer*[tiab] AND (after radiation[tiab] OR after radiotherapy[tiab] OR radiation-related[tiab] OR radiation-associated[tiab]))) AND (dose-response relationship, radiation[mh] OR radiotherapy/adverse effects[mh] OR X-ray therapy/adverse effects[mh] OR radiation injuries[mh]))

OR

(neoplasms[mh]

AND (occupational diseases[mh] OR occupational exposure[mh] OR occupation*[tiab])

AND ((worker*[tiab] OR person*[tiab]) AND (nuclear[tiab] OR radiation[tiab] OR atomic[tiab] OR weapon*[tiab])))

OR

(neoplasms[mh]

AND (((occupational diseases[mh] OR occupational exposure[mh] OR occupation*[tiab]) AND (radiation, ionizing[mh] OR radiation[tiab]))

OR occupational radiation exposure[tiab]

OR (radiation exposure[tiab] AND (clinical practice[tiab] OR medical practice[tiab]))))

OR

(neoplasms[mh]

AND (radioactive hazard release[mh] OR radioactive fallout[mh] OR nuclear warfare[mh] OR ((dose-response relationship, radiation[mh] OR radiation dose[tiab]) AND (environmental exposure[mh] OR environment[mh]))))

OR

((breast neoplasms[mh] OR (neoplasms[mh] AND breast*[tiab])) AND (background radiation[mh] OR background radiation[tiab]))

OR

((breast neoplasms[mh] OR (neoplasms, radiation-induced[mh] AND breast*[tiab]))

AND (radiography/adverse effects[mh] OR fluoroscopy/adverse effects[mh] OR radiographic examination*[tiab] OR (diagnostic[tiab] AND (radiation[tiab] OR X-ray[tiab] OR CT scan[tiab] OR computed tomography[tiab])))))

AND female[mh]

AND humans[mh]

AND (risk[mh] OR cohort studies[mh] OR incidence[mh] OR mortality[mh] OR case-control studies[mh] OR risk[tiab] OR association*[tiab] OR cancer incidence[tiab] OR cancer mortality[tiab] OR death*[tiab])

AND ("2005/01/01"[Date - Publication] : "3000"[Date - Publication])

NOT ((angiosarcoma[tiab] OR ultraviolet[tiab] OR night[tiab] OR PET[tiab] OR photon*[tiab] OR breast density[tiab] OR tolerability[tiab] OR tolerance[tiab] OR bilateral[tiab] OR synchronous[tiab] OR recurrence[tiab] OR recurrent[tiab] OR ductal carcinoma in situ[tiab]) OR (case series[tiab] OR case report[tiab] OR in vitro[tiab] OR cost-effectiveness[tiab] OR cost effective[tiab] OR sensitivity and specificity[mh:noexp] OR sensitivity and specificity[tiab] OR receiver operating characteristics[tiab] OR area under the curve[tiab]) OR (letter[ptyp] OR editorial[ptyp] OR comment[ptyp] OR news[ptyp] OR congress[ptyp] OR consensus development conference[ptyp] OR editorial[tiab] OR commentary[tiab] OR conference abstract*[tiab] OR conference proceeding*[tiab] OR systematic review*[ti] OR meta-analysis[ptyp] OR meta-analysis[ti] OR review[ptyp] OR systematic review[ptyp] OR retracted publication[ptyp] OR retraction of publication[ptyp] OR retraction of publication[tiab] OR retraction notice[ti] OR retracted publication[tiab] OR published erratum[ptyp] OR corrigenda[tiab] OR corrigendum[tiab] OR errata[tiab] OR erratum[tiab] OR protocol[ti] OR protocols[ti]) OR (animals[mh:noexp] OR mice[tiab] OR mouse[tiab] OR rat[tiab] OR rats[tiab] OR dog[tiab] OR dogs[tiab] OR pig[tiab] OR pigs[tiab] OR swine[tiab] OR porcine*[tiab] OR rodent*[tiab] OR animal*[tiab]))

*Statistical Methods*

The basis of all estimations of radiation risk is the value of ERR per unit of effective dose (ERR per Sv) or absorbed dose (ERR per Gy) of radiation exposure. The ERR is related to the relative risk (RR) by $ERR=RR-1$, so that the ERR per unit dose is $ERR/D=(RR-1)/D$ . For absorbed dose, most publications use unweighted absorbed dose (Gy), but some use weighted absorbed dose (Gy) to account for the higher biological effectiveness of neutrons compared with photons, e.g., in the LSS [2]. The basis for this in most studies is fitting of a model in which the disease/death rate (cases/deaths per year) in the group with age *a*, sex *s*, organ/tissue absorbed dose *D* (in Gy) is given by:

$\lambda(a,s)\text{ x }[1+\alpha D]$

for some function $\lambda(a,s)$ representing the disease/death rate without radiation exposure. The parameter $\alpha$ is the ERR per Gy. Additional information on maximum radiation dose, maximum radiation dose rate, age at exposure, mortality vs incidence was collected for each study endpoint within each study. When the lower confidence interval (CI) did not exist, we calculated it using the length of the upper CI, assuming a symmetric distribution of the estimate. When 90% CI $[RR_{0.05},RR_{0.95}]$ about the mean $RR_{MLE}$ was given, 95% CI was derived, assuming an approximately normal distribution of the estimate, as $[RR_{0.025},RR_{0.975}]$ where:

$$RR_{0.025}=RR_{MLE}-[RR_{MLE}-RR_{0.05}]N(0,1)_{0.975}/N(0,1)_{0.95}$$

$$RR_{0.975}=RR_{MLE}+[RR_{0.95}-RR_{MLE}]N(0,1)_{0.975}/N(0,1)_{0.95}$$

and $N(0,1)_{0.95},N(0,1)_{0.975}$ are the 95% and 97.5% centile points of a standard normal distribution, so about 1.645 and 1.960, respectively. When CIs were missing, we reconstructed the 95% CIs using the formula for the exact fiducial interval [3,4].

An aggregate estimate of ERR per Gy is computed across subsets of these studies using random-effects models and standard meta-regression methods. In certain fits in the Tables adjustment was made for certain specific factors (outcome type, exposure group, dose type, radiation dose-rate (low: <5 mGy/hour, high: ≥5 mGy/hour), maximum radiation dose, age at exposure, age at risk, concomitant chemotherapy (for studies of RT)), but in other fits given here simply the main effect of the specific factor was assessed. Random effects models were fitted by restricted maximum likelihood (REML) because of the theoretically superior performance, in particular, the absence of bias in the estimates of variance [5,6]. Confidence intervals on the meta ERR (mERR) were derived using the method of Knapp and Hartung [7]. Residual heterogeneity was assessed using Cochran’s Q-statistic. To assess the contribution of heterogeneity to the aggregate data, the Higgins and Thompson I2 statistic was computed. This is expressed as a percentage, so that a value near 0% implies little estimated inter-study heterogeneity relative to the intra-study variance, and a value near 100% implies that the inter-study heterogeneity dominates the intra-study variance [8].

To assess selection or publication bias, funnel plots were used. Funnel plots are scatterplots of the central estimates, here of ERR, against estimates of standard error, and as discussed by Egger *et al* [9,10] are useful qualitative means of detecting various types of selection bias, in particular publication bias. If the funnel plot has the form of an inverted symmetric funnel, then selection bias is considered to be unlikely [9,10]. More formal tests of selection or publication bias were also conducted using the test statistic suggested by Egger *et al* [9]. We also employed the trim-and-fill method of Duval and Tweedie [11] to assess the likely magnitude of the change in ERR that may result from selection bias.

We conducted a sensitivity analysis by sequentially excluding groups of studies based on exposure type—first removing studies of survivors of the atomic bombings, followed by those involving radiotherapy (RT)—and assessed the resulting changes in heterogeneity. Given prior concerns that the study by Cardis *et al* [12] may have led to an overestimation of pooled radiation risks [13], we performed a sensitivity analysis excluding this study to evaluate its influence on the meta-excess relative risk.

All statistical models were fitted using the metafor package [14] in R [15]. Forest plots were prepared using the forestplot package [16] in R. Results of the meta-analysis were generally based on the data given in a data supplement.

**Supplemental Table S1.1. Reasons for study exclusion.**

| **Reasons for exclusion** | **n** | **%** |
| --- | --- | --- |
| Wrong outcomes | 73 | 19 |
| No measure of association for radiation and breast cancer | 66 | 17 |
| Outcome is projected, not observed | 65 | 17 |
| No individual dose measurement (for studies of internal comparison) | 56 | 15 |
| Wrong study type | 27 | 7 |
| Wrong exposure | 26 | 7 |
| Overlapping follow-up and analysis of the same cohort | 24 | 6 |
| Wrong publication type | 15 | 4 |
| Full-text is not in English | 11 | 3 |
| No per dose analysis (i.e., estimates for given categories of dose only; for studies of internal comparison) | 7 | 2 |
| Duplicate | 4 | 1 |
| Dichotomous exposure (radiation yes/no) | 2 | 1 |
| No full text | 2 | 1 |
| No dose data (for studies of internal comparison) | 2 | 1 |
| No confidence interval or standard error for ERR | 2 | 1 |
| Not in humans | 1 | 0.3 |
| Published before 2005 | 1 | 0.3 |
| **Total** | **384** | **100** |

**Supplemental Figure S1.1A. Funnel plot for all included studies of excess relative risk (ERR).** A study with minimal publication bias should have a balanced funnel plot, with points evenly spread on both sides of the vertical line. Vertical line represents the summary effect estimate. Egger test p = 0.56. To improve visibility, studies with standard deviation > 100 (n = 3) were excluded from the plot but were retained in the meta-analysis.

**Supplemental Figure S1.1B. Funnel plot for all included studies of standardized risk ratio (SIR/SMR).** A study with minimal publication bias should have a balanced funnel plot, with points evenly spread on both sides of the vertical line. Vertical line represents the summary effect estimate. Egger test p = 0.55.

**Supplemental Table S1.2. Meta-analysis of excess relative risk for breast cancer as a result of radiation exposure, excluding studies based on exposure group to assess heterogeneity.**

|  | **No. of study endpoints** | **Meta excess relative risk/Gy (95% CI)** | **P value for residual heterogeneity^a^** | **I2 (%) (Hartung-Knapp 95% CI)^b^** |
| --- | --- | --- | --- | --- |
| **All studies** | 44 | 0.56 (0.29, 0.83) | <0.001 | 95.12 (88.27, 98.40) |
| **Excluding A-bomb studies** | 38 | 0.23 (0.10, 0.36) | <0.001 | 66.94 (32.87, 98.23) |
| **+ Excluding studies of radiation treatment for cancer^c^** | 29 | 0.44 (0.21, 0.66) | 0.11 | 21.43 (0.00, 95.49) |
| **+ Excluding studies of radiation treatment for non-cancer disease^d^** | 26 | 0.27 (0.01, 0.53) | 0.24 | 0.00 (0.00, 88.26) |
| Abbreviation: Gy – gray, CI – confidence interval.  ^a^Residual heterogeneity was assessed using Cochran’s Q statistic.  ^b^I^2^ statistic shows the proportion of total variation attributable to heterogeneity between studies vs. within studies.  ^c^Model excludes A-bomb studies and studies of radiation treatment for cancer.  ^d^Model excludes A-bomb studies and all studies of radiation treatment (i.e., for cancer and non-cancer disease). | | | | |

**Supplemental Table S1.3. Meta-analysis of higher quality estimates (mean bias score ≥3.0) of excess relative risk for breast cancer as a result of radiation exposure**

|  | **No. of study endpoints** | **Meta excess relative risk/Gy (95% CI)** | **P value for risk modification^a^** | **P value for residual heterogeneity^b^** | **I^2^ (%) (Hartung-Knapp 95% CI)^c^** |
| --- | --- | --- | --- | --- | --- |
| **ALL HIGHER QUALITY STUDIES (MEAN BIAS SCORE ≥3.0)** | | | | | |
| **Overall effect** | 26 | 0.46 (0.20, 0.73) | - | <0.001 | 94.61 (77.38, 98.02) |
| **Effect modification by outcome^d^** |  |  |  |  |  |
| Mortality | 6 | 0.48 (-0.26, 1.22) | 0.01 | <0.001 | 95.39 (79.39, 98.27) |
| Incidence | 20 | 0.48 (0.18, 0.77) |  |  |  |
| **Effect modification by exposure group (dose range)** |  |  |  |  |  |
| A-bomb studies (≤4 Gy) | 4 | 1.23 (0.78, 1.68) | <0.001 | 0.25 | 1.03 (0.00, 95.60) |
| Occupational exposure (≤5 Gy) | 9 | 0.18 (-0.11, 0.47) |  |  |  |
| Environmental exposure (≤5 Gy) | 5 | 0.07 (-1.42, 1.55) |  |  |  |
| Diagnostic exposure (≤1.7 Gy) | 1 | 2.86 (-2.18, 7.89) |  |  |  |
| Radiation treatment for cancer (≤80 Gy) | 5 | 0.05 (0.01, 0.09) |  |  |  |
| Radiation treatment for non-cancer disease (≤35 Gy) | 2 | 0.56 (0.35, 0.77) |  |  |  |
| **Effect modification by dose type (dose range)** |  |  |  |  |  |
| Breast (≤80 Gy) | 12 | 0.53 (0.21, 0.85) | 0.01 | <0.001 | 95.71 (79.54, 98.33) |
| Whole-body (≤4 Gy) | 8 | 0.16 (-0.53, 0.85) |  |  |  |
| Other (colon, stomach, unknown) (≤1 Gy) | 6 | 0.65 (-0.25, 1.55) |  |  |  |
| **Effect modification by exposure rate** |  |  |  |  |  |
| Low dose-rate, <5 mGy/hour | 16 | 0.32 (-0.19, 0.82) | 0.004 | <0.001 | 95.07 (78.46, 98.09) |
| High dose-rate, ≥5 mGy/hour | 10 | 0.54 (0.22, 0.86) |  |  |  |
| **Effect modification by maximum dose^e^** |  |  |  |  |  |
| Maximum dose ≤0.5 Gy | 8 | -0.16 (-1.71, 1.40) | 0.01 | <0.001 | 94.18 (74.43, 98.25) |
| Maximum dose >0.5 Gy to ≤1 Gy | 3 | 1.05 (-2.03, 4.13) |  |  |  |
| Maximum dose >1 Gy to ≤5 Gy | 8 | 0.78 (0.36, 1.19) |  |  |  |
| Maximum dose >5 Gy | 7 | 0.28 (-0.04, 0.60) |  |  |  |
| **Effect modification by age at exposure group^f^** |  |  |  |  |  |
| Childhood | 5 | 0.42 (0.26, 0.58) | <0.001 | 0.58 | 16.09 (0.00, 76.38) |
| Adulthood | 10 | 0.07 (-0.10, 0.25) |  |  |  |
| **Effect modification by age at risk group^g^** |  |  |  |  |  |
| 25-44 years | 3 | 0.15 (-0.02, 0.32) | 0.09 | 0.01 | 41.40 (0.00, 99.68) |
| 45-54 years | 5 | 0.04 (-0.14, 0.23) |  |  |  |
| 55-70 years | 3 | 0.53 (0.23, 0.83) |  |  |  |
| **LOW DOSE-RATE STUDIES,** **<5 mGy/hour^h^ (MEAN BIAS SCORE ≥3.0)** | | | | | |
| **Overall effect** | 16 | 0.33 (0.12, 0.54) | - | 0.66 | 13.54 (0.00, 86.38) |
| **Effect modification by dose type (dose range)** |  |  |  |  |  |
| Breast (≤80 Gy) | 3 | 0.51 (0.35, 0.68) | <0.001 | 0.87 | 0.00 (0.00, 81.00) |
| Whole-body (≤4 Gy) | 8 | 0.18 (-0.03, 0.39) |  |  |  |
| Other (colon, stomach, unknown) (≤1 Gy) | 5 | 0.09 (-1.00, 1.18) |  |  |  |
| **Effect modification by maximum dose^e^** |  |  |  |  |  |
| Maximum dose ≤0.5 Gy | 8 | -0.25 (-1.45, 0.96) | <0.001 | 0.84 | 0.00 (0.00, 88.56) |
| Maximum dose >0.5 Gy to ≤1 Gy | 3 | 1.09 (-1.55, 3.73) |  |  |  |
| Maximum dose >1 Gy to ≤5 Gy | 4 | 0.19 (-0.03, 0.41) |  |  |  |
| Maximum dose >5 Gy | 1 | 0.51 (0.35, 0.67) |  |  |  |
| **Effect modification by age at exposure group^f^** |  |  |  |  |  |
| Childhood | 2 | 0.51 (0.39, 0.64) | <0.001 | 0.96 | 0.00 (0.00, 67.71) |
| Adulthood | 9 | 0.17 (0.01, 0.35) |  |  |  |
| Abbreviation: Gy – gray, CI – confidence interval.  ^a^Each model is assessing how the excess relative risk of breast cancer per Gy of radiation exposure differs across various factors, i.e., exposure groups, radiation types, maximum doses, outcomes, age at exposure groups, and age at risk groups. Each factor was evaluated in a separate model rather than jointly.  ^b^Residual heterogeneity was assessed using Cochran’s Q statistic.  ^c^I^2^ statistic shows the proportion of total variation attributable to heterogeneity between studies vs. within studies.  ^d^Indicator variable denoting whether the study reported an incidence or mortality outcome was included to assess this effect modification.  ^e^Maximum dose reflects peak exposure rather than cumulative dose.  ^f^Only 15 studies with mean bias score ≥3.0 reported this information. Childhood exposure is defined as exposure occurring up to and including ages 18-21. Adulthood exposure is defined as exposure occurring after age 18-21.  ^g^Only 11 studies with mean bias score ≥3.0 reported this information. Age at risk, or attained age, is a study population characteristic.  ^h^The estimates for the low-dose rate studies slightly vary due to different estimation methods: ERR = 0.31 (95% CI -0.19, 0.81) is derived from a model including all high quality studies and allowing for effect modification by dose-rate, while ERR = 0.33 (95% CI 0.12, 0.53) is derived from a model including only the subgroup of higher quality low dose-rate studies. | | | | | |

**Supplemental Figure 1.2. Forest plot showing standardized risk ratios with confidence intervals for breast cancer as a result of radiation exposure, radiation treatment studies not shown.** References are shown in square brackets. Abbreviation: SIR – standardized incidence ratio, SMR – standardized mortality ratio, Dx – diagnostic exposure, A-b – studies of atomic bomb survivors.

**Supplemental Figure 1.3. Forest plot showing standardized risk ratios with confidence intervals for breast cancer as a result of radiation exposure, radiation treatment studies only.** References are shown in square brackets. Abbreviation: SIR – standardized incidence ratio, SMR – standardized mortality ratio, FU – follow-up, RT – radiation treatment, HL – Hodgkin’s lymphoma. Age at risk and FU years are reported as either means or medians, depending on data availability. [17–30][31–46][47–57][58–73][74–91]

**Supplemental Table S1.4. Characteristics of incidence and mortality studies included in the meta-analysis.**

|  | **Incidence studies n (%)** | **Mortality studies n (%)** | **P value^a^** |
| --- | --- | --- | --- |
| **Total** | 33 (100) | 11 (100) | - |
| **Exposure group** |  |  | 0.03 |
| A-bomb studies | 5 (15) | 1 (9) |  |
| Occupational exposure | 7 (21) | 8 (73) |  |
| Environmental exposure | 8 (24) | 1 (9) |  |
| Diagnostic exposure | 1 (3) | 1 (9) |  |
| Radiation treatment for cancer | 9 (28) | 0 (0) |  |
| Radiation treatment for non-cancer disease | 3 (9) | 0 (0) |  |
| **Exposure rate** |  |  | 0.04 |
| Low dose-rate, <5 mGy/hour | 18 (55) | 10 (91) |  |
| High dose-rate, ≥5 mGy/hour | 15 (45) | 1 (9) |  |
| **Maximum dose** |  |  | 0.07 |
| Maximum dose ≤0.5 Gy | 9 (27) | 6 (55) |  |
| Maximum dose >0.5 Gy to ≤1 Gy | 4 (12) | 1 (9) |  |
| Maximum dose >1 Gy to ≤5 Gy | 8 (24) | 4 (36) |  |
| Maximum dose >5 Gy | 12 (36) | 0 (0) |  |
| **Age at exposure group^b^** |  |  | 0.10 |
| Childhood | 10 (30) | 1 (9) |  |
| Adulthood | 10 (30) | 8 (73) |  |
| **Age at risk group** |  |  | 0.29 |
| 25-44 years | 5 (16) | 0 (0) |  |
| 45-54 years | 5 (16) | 0 (0) |  |
| 55-70 years | 3 (9) | 1 (9) |  |
| Abbreviation: Gy – gray.  ^a^Fisher’s Exact Test for difference in proportions between groups.  ^b^Childhood exposure is defined as exposure occurring up to and including ages 18-21. Adulthood exposure is defined as exposure occurring after age 18-21. | | | |

**Supplemental Table S1.5. Descriptive summary of additional factors from the included studies (not included in the meta-analysis)**

| **Potential risk modifier** | **Direction of effect modification** | **Study** |
| --- | --- | --- |
| **REPRODUCTIVE AND HORMONAL FACTORS** | | |
| Age at menarche | Negative | - Incidence in the Life Span Study[2] |
| Exposure around the time of menarche | Positive | - Incidence in the Life Span Study[2] |
| History of a live birth among women not treated with ovarian-damaging therapies | Positive | - RT for Hodgkin’s lymphoma[92] |
| Number of children | Positive | - US flight attendants[40] |
| Use of oral contraceptives or hormone replacement therapy | No association | - RT for Hodgkin’s lymphoma[93] |
| Age at first birth | No association | - Diagnostic X-rays for spine deformities[94] - RT for enlarged thymus[95] |
| Number of live births | No association | - RT for enlarged thymus[95] |
| Menopausal status,  Age at menopause,  Time to menopause,  Time from menopause,  Reproductive years after radiation exposure,  Nulliparous status,  Number of full-term pregnancies,  Age at first full-term pregnancy,  Years of ovarian function,  Years of ovarian function after radiation exposure | No association | - Incidence in the Life Span Study[2] |
| Menopausal status | No association | - Diagnostic X-rays for spine deformities[94] |
| **FAMILY HISTORY** | | |
| Family history of breast or ovarian cancer and radiation dose ≥5 Gy | Negative | - RT for Hodgkin’s lymphoma[92] |
| Family history of breast cancer | Positive | - Diagnostic X-rays for spine deformities[94] - Swedish hemangioma cohort[96] |
| **BIOMARKERS** | | |
| Bioavailable estradiol (bE2) | Positive, but not statistically significant | - Postmenopausal women in the Life Span Study[97] |
| Body iron levels | No association | - Postmenopausal women in the Life Span Study[98] |
| **CHEMOTHERAPY** | | |
| Anthracycline therapy | Positive | - Childhood cancer survivors[99] |
| Procarbazine dose | No association | - RT for Hodgkin’s lymphoma[93] |
| **GENETIC FACTORS** | | |
| Deleterious ATM gene variants | Positive | - Contralateral breast cancer[100] |
| **OTHER FACTORS** | | |
| BMI, smoking | No association | - Incidence in the Life Span Study[2] |
| Family income | No association | - Diagnostic X-rays for spine deformities[94] |

**SUPPLEMENTAL DISCUSSION**

**Additional topics in radiation exposure studies**

*Complexities in assessing subsequent cancer risk following radiotherapy*

The development of subsequent malignancies in populations exposed to radiotherapy involves numerous complexities that extend beyond radiation exposure alone. Several strong risk factors contribute to this risk, including shared etiological factors between the primary and subsequent cancers (e.g., breast cancer with thyroid or endometrial cancers) [101,102]. as well as exposure to specific chemotherapeutic agents such as anthracyclines [103,104]. Accurately isolating the effect of RT on the development of subsequent breast cancer requires careful consideration of these confounding variables.

Moreover, methodological limitations further complicate this area of study. These include:

1) The strong implicit assumption that primary and subsequent cancers are biologically independent events,
2) The influence of medical surveillance bias, wherein increased monitoring of cancer survivors may lead to higher detection rates (which, in part, lead to earlier diagnosis) of subsequent cancers compared to the general population [105].

*Radiation exposure during breast development*

A previous non-systematic review explored the long-standing hypothesis that the breast is particularly susceptible to carcinogenic exposures during periods of active development. However, until 2004, no studies with sufficient statistical power to evaluate this hypothesis had been identified. In the two decades since, three large cohort studies involving individuals exposed to ionizing radiation during childhood and adolescence—one of which met the inclusion criteria for this review [2]—have provided consistent evidence supporting this hypothesis. These studies [2,60,106] demonstrate that the excess relative risk per gray (ERR/Gy) peaks during a relatively narrow window around menarche, a period likely serving as a proxy for the rapid breast growth and cellular proliferation characteristic of puberty.

*Limited Evidence from Diagnostic Radiation Studies*

This review includes only two reports, both stemming from a single study on diagnostic radiation exposure [56,94]. The primary reason for this limited inclusion is that large-scale epidemiological studies on computed tomography scans have not yet been able to assess breast cancer risk adequately. This limitation is largely due to insufficient follow-up time and the consequently low expected number of (excess) breast cancer cases in young populations. To date, existing studies have primarily evaluated tumors arising at younger ages, for which a clear dose-response relationship at low radiation doses is still anticipated. Assessments of breast cancer risk, however, rely on projections rather than observed cases [107].

**References:**

1	United Nations Scientific Committee on the Effects of Atomic, Radiation (UNSCEAR). *UNSCEAR 2006 Report. Annex A. Epidemiological Studies of Radiation and Cancer*. United Nations 2008.

2	Brenner AV, Preston DL, Sakata R, *et al.* Incidence of Breast Cancer in the Life Span Study of Atomic Bomb Survivors: 1958-2009. *Radiat Res*. 2018;190:433–44. doi: 10.1667/RR15015.1

3	Thulin Må, Zwanzig S. Exact confidence intervals and hypothesis tests for parameters of discrete distributions. *Bernoulli*. 2017;23. doi: 10.3150/15-BEJ750

4	Wang YH. Fiducial Intervals: What are They? *The American Statistician*. 2000;54:105–11. doi: 10.1080/00031305.2000.10474520

5	Viechtbauer W. Bias and Efficiency of Meta-Analytic Variance Estimators in the Random-Effects Model. *Journal of Educational and Behavioral Statistics*. 2005;30:261–93. doi: 10.3102/10769986030003261

6	Bartlett MS, Fowler RH. Properties of sufficiency and statistical tests. *Proceedings of the Royal Society of London Series A - Mathematical and Physical Sciences*. 1937;160:268–82. doi: 10.1098/rspa.1937.0109

7	Knapp G, Hartung J. Improved tests for a random effects meta-regression with a single covariate. *Stat Med*. 2003;22:2693–710. doi: 10.1002/sim.1482

8	Higgins JPT, Thompson SG. Quantifying heterogeneity in a meta-analysis. *Stat Med*. 2002;21:1539–58. doi: 10.1002/sim.1186

9	Egger M, Davey Smith G, Schneider M, *et al.* Bias in meta-analysis detected by a simple, graphical test. *BMJ*. 1997;315:629–34. doi: 10.1136/bmj.315.7109.629

10	Sterne JA, Egger M. Funnel plots for detecting bias in meta-analysis: guidelines on choice of axis. *J Clin Epidemiol*. 2001;54:1046–55. doi: 10.1016/s0895-4356(01)00377-8

11	Duval S, Tweedie R. A Nonparametric “Trim and Fill” Method of Accounting for Publication Bias in Meta-Analysis. *Journal of the American Statistical Association*. 2000;95:89–98. doi: 10.1080/01621459.2000.10473905

12	Cardis E, Vrijheid M, Blettner M, *et al.* The 15-Country Collaborative Study of Cancer Risk among Radiation Workers in the Nuclear Industry: estimates of radiation-related cancer risks. *Radiat Res*. 2007;167:396–416. doi: 10.1667/RR0553.1

13	Zablotska LB, Lane RSD, Thompson PA. A reanalysis of cancer mortality in Canadian nuclear workers (1956–1994) based on revised exposure and cohort data. *Br J Cancer*. 2014;110:214–23. doi: 10.1038/bjc.2013.592

14	Viechtbauer W. Conducting Meta-Analyses in R with the metafor Package. *Journal of Statistical Software*. 2010;36:1–48. doi: 10.18637/jss.v036.i03

15	R Core Team. R: A Language and Environment for Statistical Computing. R Foundation for Statistical Computing, Vienna, Austria. <https://www.R-project.org/>. 2024.

16	Gordon M, Lumley T. forestplot: Advanced Forest Plot Using ‘grid’ Graphics. R package version 3.1.7 <https://CRAN.R-project.org/package=forestplot>. 2025.

17	Goto H, Watanabe T, Miyao M, *et al.* Cancer mortality among atomic bomb survivors exposed as children. *Environ Health Prev Med*. 2012;17:228–34. doi: 10.1007/s12199-011-0246-6

18	Watanabe T, Miyao M, Honda R, *et al.* Hiroshima survivors exposed to very low doses of A-bomb primary radiation showed a high risk for cancers. *Environ Health Prev Med*. 2008;13:264–70. doi: 10.1007/s12199-008-0039-8

19	Habib RR, Abdallah SM, Law M, *et al.* Mortality rates among nuclear industry workers at Lucas Heights Science and Technology Centre. *Aust N Z J Public Health*. 2005;29:229–37. doi: 10.1111/j.1467-842x.2005.tb00760.x

20	Martin S, Ségala C. Epidemiological Study of Mortality Among Workers Exposed to Tritium in France. *Radiat Res*. 2021;195:284–92. doi: 10.1667/RR15353.1

21	Jartti P, Pukkala E, Uitti J, *et al.* Cancer incidence among physicians occupationally exposed to ionizing radiation in Finland. *Scand J Work Environ Health*. 2006;32:368–73. doi: 10.5271/sjweh.1032

22	Habib RR, Abdallah SM, Law M, *et al.* Cancer incidence among Australian nuclear industry workers. *J Occup Health*. 2006;48:358–65. doi: 10.1539/joh.48.358

23	Park S, Seo S, Lee D, *et al.* A Cohort Study of Korean Radiation Workers: Baseline Characteristics of Participants. *Int J Environ Res Public Health*. 2020;17:2328. doi: 10.3390/ijerph17072328

24	Pukkala E, Helminen M, Haldorsen T, *et al.* Cancer incidence among Nordic airline cabin crew. *Int J Cancer*. 2012;131:2886–97. doi: 10.1002/ijc.27551

25	Zhivin S, Guseva Canu I, Samson E, *et al.* Mortality (1968-2008) in a French cohort of uranium enrichment workers potentially exposed to rapidly soluble uranium compounds. *Occup Environ Med*. 2016;73:167–74. doi: 10.1136/oemed-2015-103142

26	Schubauer-Berigan MK, Anderson JL, Hein MJ, *et al.* Breast cancer incidence in a cohort of U.S. flight attendants. *Am J Ind Med*. 2015;58:252–66. doi: 10.1002/ajim.22419

27	Guseva Canu I, Rogel A, Samson E, *et al.* Cancer mortality risk among biology research workers in France: first results of two retrospective cohorts studies. *Int Arch Occup Environ Health*. 2008;81:777–85. doi: 10.1007/s00420-007-0260-6

28	Samson E, Piot I, Zhivin S, *et al.* Cancer and non-cancer mortality among French uranium cycle workers: the TRACY cohort. *BMJ Open*. 2016;6:e010316. doi: 10.1136/bmjopen-2015-010316

29	Samson E, Telle-Lamberton M, Caër-Lorho S, *et al.* Cancer mortality among two different populations of French nuclear workers. *Int Arch Occup Environ Health*. 2011;84:627–34. doi: 10.1007/s00420-011-0636-5

30	Silver SR, Bertke SJ, Hein MJ, *et al.* Mortality and ionising radiation exposures among workers employed at the Fernald Feed Materials Production Center (1951-1985). *Occup Environ Med*. 2013;70:453–63. doi: 10.1136/oemed-2012-100768

31	Lee WJ, Ko S, Bang YJ, *et al.* Occupational radiation exposure and cancer incidence in a cohort of diagnostic medical radiation workers in South Korea. *Occup Environ Med*. 2021;78:876–83. doi: 10.1136/oemed-2021-107452

32	Ringen K, Dement J, Hines S, *et al.* Mortality of older construction and craft workers employed at department of energy nuclear sites: Follow-up through 2016. *Am J Ind Med*. 2019;62:742–54. doi: 10.1002/ajim.23018

33	Engels H, Swaen GMH, Slangen J, *et al.* Radiation exposure and cause specific mortality among nuclear workers in Belgium (1969-1994). *Radiat Prot Dosimetry*. 2005;117:373–81. doi: 10.1093/rpd/nci327

34	Lee WJ, Ha M, Hwang S, *et al.* The radiologic technologists’ health study in South Korea: study design and baseline results. *Int Arch Occup Environ Health*. 2015;88:759–68. doi: 10.1007/s00420-014-1002-1

35	Preston DL, Kitahara CM, Freedman DM, *et al.* Breast cancer risk and protracted low-to-moderate dose occupational radiation exposure in the US Radiologic Technologists Cohort, 1983-2008. *Br J Cancer*. 2016;115:1105–12. doi: 10.1038/bjc.2016.292

36	Dreger S, Wollschläger D, Schafft T, *et al.* Cohort study of occupational cosmic radiation dose and cancer mortality in German aircrew, 1960-2014. *Occup Environ Med*. 2020;77:285–91. doi: 10.1136/oemed-2019-106165

37	Milacic S. The incidence of malignant neoplasms in individuals working in areas of ionizing radiation in hospitals. *J BUON*. 2008;13:377–84.

38	Metz-Flamant C, Samson E, Caër-Lorho S, *et al.* Solid cancer mortality associated with chronic external radiation exposure at the French atomic energy commission and nuclear fuel company. *Radiat Res*. 2011;176:115–27. doi: 10.1667/RR2528.1

39	Boice JD, Cohen SS, Mumma MT, *et al.* Mortality among mound workers exposed to polonium-210 and other sources of radiation, 1944-1979. *Radiat Res*. 2014;181:208–28. doi: 10.1667/RR13395.1

40	Pinkerton LE, Hein MJ, Anderson JL, *et al.* Breast cancer incidence among female flight attendants: exposure-response analyses. *Scand J Work Environ Health*. 2016;42:538–46. doi: 10.5271/sjweh.3586

41	Zielinski JM, Garner MJ, Band PR, *et al.* Health outcomes of low-dose ionizing radiation exposure among medical workers: a cohort study of the Canadian national dose registry of radiation workers. *Int J Occup Med Environ Health*. 2009;22:149–56. doi: 10.2478/v10001-009-0010-y

42	Boice JD, Cohen SS, Mumma MT, *et al.* Updated mortality analysis of radiation workers at Rocketdyne (Atomics International), 1948-2008. *Radiat Res*. 2011;176:244–58. doi: 10.1667/RR2487.1

43	Yiin JH, Anderson JL, Daniels RD, *et al.* Mortality in a combined cohort of uranium enrichment workers. *Am J Ind Med*. 2017;60:96–108. doi: 10.1002/ajim.22668

44	Samerdokiene V, Atkocius V, Valuckas KP. The risk of cancer among Lithuanian medical radiation workers in 1978-2004. *Medicina (Kaunas)*. 2009;45:412–8.

45	Richardson DB, Wing S, Keil A, *et al.* Mortality among workers at Oak Ridge National Laboratory. *Am J Ind Med*. 2013;56:725–32. doi: 10.1002/ajim.22164

46	Lee WJ, Ko S, Bang YJ, *et al.* Mortality among diagnostic medical radiation workers in South Korea, 1996-2015. *Occup Environ Med*. 2018;75:739–41. doi: 10.1136/oemed-2018-105019

47	Bouet S, Davesne E, Samson E, *et al.* Analysis of the association between ionizing radiation and mortality in uranium workers from five plants involved in the nuclear fuel production cycle in France. *Int Arch Occup Environ Health*. 2019;92:249–62. doi: 10.1007/s00420-018-1375-7

48	Gillies M, Haylock R. The cancer mortality and incidence experience of workers at British Nuclear Fuels plc, 1946-2005. *J Radiol Prot*. 2014;34:595–623. doi: 10.1088/0952-4746/34/3/595

49	Kubale T, Hiratzka S, Henn S, *et al.* A cohort mortality study of chemical laboratory workers at Department of Energy Nuclear Plants. *Am J Ind Med*. 2008;51:656–67. doi: 10.1002/ajim.20601

50	Prysyazhnyuk A, Gristchenko V, Fedorenko Z, *et al.* Twenty years after the Chernobyl accident: solid cancer incidence in various groups of the Ukrainian population. *Radiat Environ Biophys*. 2007;46:43–51. doi: 10.1007/s00411-007-0093-4

51	Boice JD, Cohen SS, Mumma MT, *et al.* Mortality among residents of Uravan, Colorado who lived near a uranium mill, 1936-84. *J Radiol Prot*. 2007;27:299–319. doi: 10.1088/0952-4746/27/3/004

52	Bazyka D, Prysyazhnyuk A, Gudzenko N, *et al.* Epidemiology of Late Health Effects in Ukrainian Chornobyl Cleanup Workers. *Health Phys*. 2018;115:161–9. doi: 10.1097/HP.0000000000000868

53	Hwang S-L, Guo H-R, Hsieh W-A, *et al.* Cancer risks in a population with prolonged low dose-rate gamma-radiation exposure in radiocontaminated buildings, 1983-2002. *Int J Radiat Biol*. 2006;82:849–58. doi: 10.1080/09553000601085980

54	Kurttio P, Pukkala E, Ilus T, *et al.* Radiation doses from global fallout and cancer incidence among reindeer herders and Sami in Northern Finland. *Occup Environ Med*. 2010;67:737–43. doi: 10.1136/oem.2009.048652

55	Ostroumova E, Preston DL, Ron E, *et al.* Breast cancer incidence following low-dose rate environmental exposure: Techa River Cohort, 1956-2004. *Br J Cancer*. 2008;99:1940–5. doi: 10.1038/sj.bjc.6604775

56	Ronckers CM, Land CE, Miller JS, *et al.* Cancer mortality among women frequently exposed to radiographic examinations for spinal disorders. *Radiat Res*. 2010;174:83–90. doi: 10.1667/RR2022.1

57	Evans DG, Kotre CJ, Harkness E, *et al.* No strong evidence for increased risk of breast cancer 8-26 years after multiple mammograms in their 30s in females at moderate and high familial risk. *Br J Radiol*. 2016;89:20150960. doi: 10.1259/bjr.20150960

58	O’Brien MM, Donaldson SS, Balise RR, *et al.* Second malignant neoplasms in survivors of pediatric Hodgkin’s lymphoma treated with low-dose radiation and chemotherapy. *J Clin Oncol*. 2010;28:1232–9. doi: 10.1200/JCO.2009.24.8062

59	Lange JM, Takashima JR, Peterson SM, *et al.* Breast cancer in female survivors of Wilms tumor: a report from the national Wilms tumor late effects study. *Cancer*. 2014;120:3722–30. doi: 10.1002/cncr.28908

60	Moskowitz CS, Chou JF, Sklar CA, *et al.* Radiation-associated breast cancer and gonadal hormone exposure: a report from the Childhood Cancer Survivor Study. *Br J Cancer*. 2017;117:290–9. doi: 10.1038/bjc.2017.169

61	Castellino SM, Geiger AM, Mertens AC, *et al.* Morbidity and mortality in long-term survivors of Hodgkin lymphoma: a report from the Childhood Cancer Survivor Study. *Blood*. 2011;117:1806–16. doi: 10.1182/blood-2010-04-278796

62	Guibout C, Adjadj E, Rubino C, *et al.* Malignant breast tumors after radiotherapy for a first cancer during childhood. *J Clin Oncol*. 2005;23:197–204. doi: 10.1200/JCO.2005.06.225

63	Inskip PD, Robison LL, Stovall M, *et al.* Radiation dose and breast cancer risk in the childhood cancer survivor study. *J Clin Oncol*. 2009;27:3901–7. doi: 10.1200/JCO.2008.20.7738

64	Taylor AJ, Winter DL, Stiller CA, *et al.* Risk of breast cancer in female survivors of childhood Hodgkin’s disease in Britain: a population-based study. *Int J Cancer*. 2007;120:384–91. doi: 10.1002/ijc.22261

65	Travis LB, Hill DA, Dores GM, *et al.* Breast cancer following radiotherapy and chemotherapy among young women with Hodgkin disease. *JAMA*. 2003;290:465–75. doi: 10.1001/jama.290.4.465

66	Alm El-Din MA, Hughes KS, Finkelstein DM, *et al.* Breast cancer after treatment of Hodgkin’s lymphoma: risk factors that really matter. *Int J Radiat Oncol Biol Phys*. 2009;73:69–74. doi: 10.1016/j.ijrobp.2008.03.066

67	Turcotte LM, Whitton JA, Friedman DL, *et al.* Risk of Subsequent Neoplasms During the Fifth and Sixth Decades of Life in the Childhood Cancer Survivor Study Cohort. *J Clin Oncol*. 2015;33:3568–75. doi: 10.1200/JCO.2015.60.9487

68	De Bruin ML, Sparidans J, van’t Veer MB, *et al.* Breast cancer risk in female survivors of Hodgkin’s lymphoma: lower risk after smaller radiation volumes. *J Clin Oncol*. 2009;27:4239–46. doi: 10.1200/JCO.2008.19.9174

69	Petrakova K, Vyskocil J, Grell P, *et al.* Second cancers in Hodgkin’s lymphoma long-term survivals: A 60-year single institutional experience with real-life cohort of 871 patients. *Int J Clin Pract*. 2018;e13235. doi: 10.1111/ijcp.13235

70	Schaapveld M, Aleman BMP, van Eggermond AM, *et al.* Second Cancer Risk Up to 40 Years after Treatment for Hodgkin’s Lymphoma. *N Engl J Med*. 2015;373:2499–511. doi: 10.1056/NEJMoa1505949

71	Dores GM, Anderson WF, Beane Freeman LE, *et al.* Risk of breast cancer according to clinicopathologic features among long-term survivors of Hodgkin’s lymphoma treated with radiotherapy. *Br J Cancer*. 2010;103:1081–4. doi: 10.1038/sj.bjc.6605877

72	Swerdlow AJ, Cooke R, Bates A, *et al.* Breast cancer risk after supradiaphragmatic radiotherapy for Hodgkin’s lymphoma in England and Wales: a National Cohort Study. *J Clin Oncol*. 2012;30:2745–52. doi: 10.1200/JCO.2011.38.8835

73	Kleinerman RA, Tucker MA, Tarone RE, *et al.* Risk of new cancers after radiotherapy in long-term survivors of retinoblastoma: an extended follow-up. *J Clin Oncol*. 2005;23:2272–9. doi: 10.1200/JCO.2005.05.054

74	Veit-Rubin N, Rapiti E, Usel M, *et al.* Risk, characteristics, and prognosis of breast cancer after Hodgkin’s lymphoma. *Oncologist*. 2012;17:783–91. doi: 10.1634/theoncologist.2011-0451

75	Berrington de Gonzalez A, Curtis RE, Gilbert E, *et al.* Second solid cancers after radiotherapy for breast cancer in SEER cancer registries. *Br J Cancer*. 2010;102:220–6. doi: 10.1038/sj.bjc.6605435

76	Reulen RC, Taylor AJ, Winter DL, *et al.* Long-term population-based risks of breast cancer after childhood cancer. *Int J Cancer*. 2008;123:2156–63. doi: 10.1002/ijc.23743

77	Swerdlow AJ, Barber JA, Hudson GV, *et al.* Risk of second malignancy after Hodgkin’s disease in a collaborative British cohort: the relation to age at treatment. *J Clin Oncol*. 2000;18:498–509. doi: 10.1200/JCO.2000.18.3.498

78	Bartkowiak D, Humble N, Suhr P, *et al.* Second cancer after radiotherapy, 1981-2007. *Radiother Oncol*. 2012;105:122–6. doi: 10.1016/j.radonc.2011.09.013

79	Fridman M, Krasko O, Levin L, *et al.* Second primary malignancies in patients with papillary thyroid carcinoma after effect of post-chernobyl irradiation: A risk analysis of more than two decades of observations. *Cancer Epidemiol*. 2021;70:101860. doi: 10.1016/j.canep.2020.101860

80	Chow JCH, Au KH, Mang OWK, *et al.* Risk, pattern and survival impact of second primary tumors in patients with nasopharyngeal carcinoma following definitive intensity-modulated radiotherapy. *Asia Pac J Clin Oncol*. 2019;15:48–55. doi: 10.1111/ajco.12994

81	Levi F, Randimbison L, Te V-C, *et al.* Cancer risk after radiotherapy for breast cancer. *Br J Cancer*. 2006;95:390–2. doi: 10.1038/sj.bjc.6603235

82	Hamilton SN, Tyldesley S, Li D, *et al.* Second malignancies after adjuvant radiation therapy for early stage breast cancer: is there increased risk with addition of regional radiation to local radiation? *Int J Radiat Oncol Biol Phys*. 2015;91:977–85. doi: 10.1016/j.ijrobp.2014.12.051

83	McDonald AM, Chen Y, Wu J, *et al.* Total Body Irradiation and Risk of Breast Cancer After Blood or Marrow Transplantation: A Blood or Marrow Transplantation Survivor Study Report. *J Clin Oncol*. 2020;38:2872–82. doi: 10.1200/JCO.20.00231

84	Brown AP, Chen J, Hitchcock YJ, *et al.* The risk of second primary malignancies up to three decades after the treatment of differentiated thyroid cancer. *J Clin Endocrinol Metab*. 2008;93:504–15. doi: 10.1210/jc.2007-1154

85	Tward JD, Wendland MMM, Shrieve DC, *et al.* The risk of secondary malignancies over 30 years after the treatment of non-Hodgkin lymphoma. *Cancer*. 2006;107:108–15. doi: 10.1002/cncr.21971

86	Kumar S, Shah JP, Bryant CS, *et al.* Second neoplasms in survivors of endometrial cancer: impact of radiation therapy. *Gynecol Oncol*. 2009;113:233–9. doi: 10.1016/j.ygyno.2008.12.039

87	Canchola AJ, Horn-Ross PL, Purdie DM. Risk of second primary malignancies in women with papillary thyroid cancer. *Am J Epidemiol*. 2006;163:521–7. doi: 10.1093/aje/kwj072

88	Nekolla EA, Walsh L, Spiess H. Incidence of malignant diseases in humans injected with radium-224. *Radiat Res*. 2010;174:377–86. doi: 10.1667/RR1955.1

89	Haddy N, Dondon M-G, Paoletti C, *et al.* Breast cancer following radiotherapy for a hemangioma during childhood. *Cancer Causes Control*. 2010;21:1807–16. doi: 10.1007/s10552-010-9607-5

90	Antunes L, Bento MJ, Sobrinho-Simões M, *et al.* Cancer incidence after childhood irradiation for tinea capitis in a Portuguese cohort. *Br J Radiol*. 2020;93:20180677. doi: 10.1259/bjr.20180677

91	Sakata R, Kleinerman RA, Mabuchi K, *et al.* Cancer mortality following radiotherapy for benign gynecologic disorders. *Radiat Res*. 2012;178:266–79. doi: 10.1667/rr2845.1

92	Hill DA, Gilbert E, Dores GM, *et al.* Breast cancer risk following radiotherapy for Hodgkin lymphoma: modification by other risk factors. *Blood*. 2005;106:3358–65. doi: 10.1182/blood-2005-04-1535

93	Krul IM, Opstal-van Winden AWJ, Aleman BMP, *et al.* Breast Cancer Risk After Radiation Therapy for Hodgkin Lymphoma: Influence of Gonadal Hormone Exposure. *Int J Radiat Oncol Biol Phys*. 2017;99:843–53. doi: 10.1016/j.ijrobp.2017.07.016

94	Ronckers CM, Doody MM, Lonstein JE, *et al.* Multiple diagnostic X-rays for spine deformities and risk of breast cancer. *Cancer Epidemiol Biomarkers Prev*. 2008;17:605–13. doi: 10.1158/1055-9965.EPI-07-2628

95	Adams MJ, Dozier A, Shore RE, *et al.* Breast cancer risk 55+ years after irradiation for an enlarged thymus and its implications for early childhood medical irradiation today. *Cancer Epidemiol Biomarkers Prev*. 2010;19:48–58. doi: 10.1158/1055-9965.EPI-09-0520

96	Eidemüller M, Holmberg E, Lundell M, *et al.* Evidence for Increased Susceptibility to Breast Cancer From Exposure to Ionizing Radiation Due to a Familial History of Breast Cancer: Results From the Swedish Hemangioma Cohort. *Am J Epidemiol*. 2021;190:76–84. doi: 10.1093/aje/kwaa163

97	Grant EJ, Cologne ,John B., Sharp ,Gerald B., *et al.* Bioavailable serum estradiol may alter radiation risk of postmenopausal breast cancer: a nested case-control study. *International Journal of Radiation Biology*. 2018;94:97–105. doi: 10.1080/09553002.2018.1419303

98	Stevens RG, Cologne JB, Nakachi K, *et al.* Body iron stores and breast cancer risk in female atomic bomb survivors. *Cancer Science*. 2011;102:2236–40. doi: 10.1111/j.1349-7006.2011.02080.x

99	Veiga LH, Curtis RE, Morton LM, *et al.* Association of Breast Cancer Risk After Childhood Cancer With Radiation Dose to the Breast and Anthracycline Use: A Report From the Childhood Cancer Survivor Study. *JAMA Pediatr*. 2019;173:1171–9. doi: 10.1001/jamapediatrics.2019.3807

100	Bernstein JL, Haile RW, Stovall M, *et al.* Radiation Exposure, the ATM Gene, and Contralateral Breast Cancer in the Women’s Environmental Cancer and Radiation Epidemiology Study. *JNCI: Journal of the National Cancer Institute*. 2010;102:475–83. doi: 10.1093/jnci/djq055

101	Nielsen SM, White MG, Hong S, *et al.* The Breast-Thyroid Cancer Link: A Systematic Review and Meta-analysis. *Cancer Epidemiol Biomarkers Prev*. 2016;25:231–8. doi: 10.1158/1055-9965.EPI-15-0833

102	Lai Y-L, Chiang C-J, Chen Y-L, *et al.* Increased risk of second primary malignancies among endometrial cancer survivors receiving surgery alone: A population-based analysis. *Cancer Medicine*. 2021;10:6845–54. doi: 10.1002/cam4.3861

103	Wang Y, Ronckers CM, van Leeuwen FE, *et al.* Subsequent female breast cancer risk associated with anthracycline chemotherapy for childhood cancer. *Nat Med*. 2023;29:2268–77. doi: 10.1038/s41591-023-02514-1

104	van Leeuwen FE, Ronckers CM. Anthracyclines and Alkylating Agents: New Risk Factors for Breast Cancer in Childhood Cancer Survivors? *J Clin Oncol*. 2016;34:891–4. doi: 10.1200/JCO.2015.65.0465

105	Curtis RE, Freedman DM, Ron E, *et al.* Methods. *New Malignancies Among Cancer Survivors: SEER Cancer Registries, 1973-2000*. Bethesda, MD: National Cancer Institute, NIH Publ. 2006.

106	Cooke R, Jones ME, Cunningham D, *et al.* Breast cancer risk following Hodgkin lymphoma radiotherapy in relation to menstrual and reproductive factors. *Br J Cancer*. 2013;108:2399–406. doi: 10.1038/bjc.2013.219

107	Smith-Bindman R, Chu PW, Azman Firdaus H, *et al.* Projected Lifetime Cancer Risks From Current Computed Tomography Imaging. *JAMA Intern Med*. 2025;185:710–9. doi: 10.1001/jamainternmed.2025.0505
